# The WNK1–ERK5 route plays a pathophysiological role in ovarian cancer and limits therapeutic efficacy of trametinib

**DOI:** 10.1002/ctm2.1217

**Published:** 2023-04-08

**Authors:** Adrián Sánchez‐Fdez, Sofía Matilla‐Almazán, Juan Carlos Montero, Sofía Del Carmen, Mar Abad, Sara García‐Alonso, Somshuvra Bhattacharya, Kristin Calar, Pilar de la Puente, Alberto Ocaña, Atanasio Pandiella, Azucena Esparís‐Ogando

**Affiliations:** ^1^ Instituto de Biología Molecular y Celular del Cáncer‐CSIC Salamanca Spain; ^2^ Instituto de Investigación Biomédica de Salamanca (IBSAL) Salamanca Spain; ^3^ Centro de Investigación Biomédica en Red de Cáncer (CIBERONC) Salamanca Spain; ^4^ Moores Cancer Center, University of California San Diego La Jolla CA USA; ^5^ Department of Pathology University Hospital of Salamanca Salamanca Spain; ^6^ Cancer Biology and Immunotherapies Group Sanford Research Sioux Falls South Dakota USA; ^7^ Department of Surgery Sanford School of Medicine, University of South Dakota Sioux Falls South Dakota USA; ^8^ Experimental Therapeutics Unit CRIS‐Cancer Hospital Clínico San Carlos and CIBERONC Madrid Spain; ^9^ Universidad de Castilla La Mancha, Castilla La Mancha Albacete Spain

**Keywords:** ERK5, MEK5, ovarian cancer, patient biopsies, trametinib, WNK1

## Abstract

**Background:**

The dismal prognosis of advanced ovarian cancer calls for the development of novel therapies to improve disease outcome. In this regard, we set out to discover new molecular entities and to assess the preclinical effectiveness of their targeting.

**Methods:**

Cell lines, mice and human ovarian cancer samples were used. Proteome profiling of human phosphokinases, in silico genomic analyses, genetic (shRNA and CRISPR/Cas9) and pharmacological strategies as well as an ex vivo human preclinical model were performed.

**Results:**

We identified WNK1 as a highly phosphorylated protein in ovarian cancer and found that its activation or high expression had a negative impact on patients’ survival. Genomic analyses showed amplification of *WNK1* in human ovarian tumours. Mechanistically, we demonstrate that WNK1 exerted its action through the MEK5–ERK5 signalling module in ovarian cancer. Loss of function, genetic or pharmacological experiments, demonstrated anti‐proliferative and anti‐tumoural effects of the targeting of the WNK1–MEK5–ERK5 route. Additional studies showed that this pathway modulated the anti‐tumoural properties of the MEK1/2 inhibitor trametinib. Thus, treatment with trametinib activated the WNK1–MEK5–ERK5 route, raising the possibility that this effect may limit the therapeutic benefit of ERK1/2 targeting in ovarian cancer. Moreover, in different experimental settings, including an ex vivo patient‐derived model consisting of ovarian cancer cells cultured with autologous patient sera, we show that inhibition of WNK1 or MEK5 increased the anti‐proliferative and anti‐tumour efficacy of trametinib.

**Conclusions:**

The present study uncovers the participation of WNK1–MEK5–ERK5 axis in ovarian cancer pathophysiology, opening the possibility of acting on this pathway with therapeutic purposes. Another important finding of the present study was the activation of that signalling axis by trametinib, bypassing the anti‐tumoural efficacy of this drug. That fact should be considered in the context of the use of trametinib in ovarian cancer.

## Graphical abstract

1. Activation of WNK1 associates to a worse ovarian cancer patient outcome.
2. WNK1 signals cell proliferation through the ERK5 pathway.
3. Trametinib provokes activation of the WNK1–ERK5 pathway.
4. Combining trametinib with ERK5 route inhibitors may improve patients’ outcome.

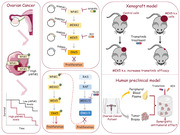

## 1 BACKGROUND

Every year, 295,000 people are diagnosed with ovarian cancer, and the number of deaths caused by this disease reaches 185,000.^1^ The therapy of these tumours includes surgical resection as well as medical treatment, which is classically based on chemotherapeutic compounds.^2^ More recently, inhibitors of poly‐ADP ribose polymerase or the anti‐angiogenic antibody bevacizumab have also shown efficacy in ovarian cancer.^3^, ^4^, ^5^, ^6^ Additional strategies under clinical evaluation include the use of immune checkpoint inhibitors or antibody–drug conjugates targeting cell surface proteins.^7^, ^8^, ^9^ Despite these therapeutic advances, the disseminated disease remains incurable. Due to this, efforts to increase knowledge on the pathophysiological entities that contribute to tumour generation/progression continue to being carried out. Identification of such key molecules may enable the development of targeted therapies that could help in the management of the disease.

Mitogen‐activate protein kinases (MAPKs) play important roles as signal transducers in mediating proliferative responses.^10^ In mammals, four major MAPK routes have been described, including the ERK1/2, p38, JNK and the ERK5 pathways.^10^ These routes are typically organised into kinase cascades, consisting of a MAPK kinase kinase, a MAPK kinase or MEK and the MAPK. Their critical role in the transduction of physiological signals related to cell proliferation has fostered their study as targets for therapeutic intervention in cancer. Moreover, their altered signalling in certain tumoural diseases has promoted the development of agents that have been approved for clinical use. That is the case of the classical ERK1/2 route in melanoma. In that disease, the constitutive activation of mutated BRAF kinase provokes activation of the ERK1/2 route, and this contributes to unregulated proliferation of melanoma cells.^11^ Agents such as trametinib or cobimetinib, that inhibit the activity of MEK1/2, the ERK1/2 upstream activating kinases, have been approved for patients with BRAF‐mutant melanomas.^12^ In ovarian cancer, several preclinical studies have suggested that the ERK1/2 route may contribute to the development of the disease.^13^, ^14^ Because of that, clinical studies using the MEK1/2 kinase inhibitor trametinib have been performed or are ongoing. Some of these studies showed benefit of therapeutic regimes using trametinib.^15^

In addition to the ERK1/2 route, the ERK5 pathway has also attracted substantial interest as a mediator of proliferative responses. Molecular components of this pathway include ERK5 and its upstream activating kinase MEK5.^16^ The latter is activated by MEKK2 or MEKK3, which in turn may be stimulated by other upstream proteins such as Cot1 or WNK1.^17^, ^18^ This route may be activated by growth factor receptors as well as by other stimuli, such as changes in extracellular osmotic pressure.^19^, ^20^ In vitro as well as in vivo studies have demonstrated a critical role of the ERK5 route in cell proliferation and animal development.^21^, ^22^ Moreover, a pathophysiological role of this kinase pathway in tumorigenesis has recently been demonstrated using mice expressing MEK5DD, a constitutively active form of MEK5.^23^ Transgenic expression of MEK5DD caused constitutive activation of ERK5 and the development of lung adenocarcinomas.^23^ In addition, several in vitro studies suggested that this kinase route also plays a role in the control of proliferation of several tumoural cell types.^24^ However, a detailed analysis of the impact of the ERK5 pathway in ovarian cancer tumorigenesis has not been performed.

Here we show that the WNK1–MEK5–ERK5 module plays a role in the proliferation of ovarian cancer cells. Moreover, we show that targeting the ERK1/2 route provoked activation of the WNK1–ERK5 pathway, and this hampered the anti‐proliferative effect of the MEK1/2 inhibitor trametinib. In vitro and in vivo experiments, complemented with ex vivo studies using primary ovarian tumour samples cultured within an autologous human plasma 3D model (HuP3D), demonstrated the superior anti‐proliferative effect of the targeting of both routes simultaneously. Therefore, and given the clinical interest in the use of ERK1/2 inhibitors, our data indicate that to avoid the activation of WNK1–ERK5 after trametinib treatment and consequently to achieve an optimal anti‐tumoural effect, the targeting of both ERK1/2 and WNK1–ERK5 routes should be taken into consideration. In the case of ovarian cancer, these findings acquire especial relevance since a recent clinical trial demonstrated the anti‐tumoural efficacy of trametinib in this disease.^15^

## 2 METHODS

### 2.1 Reagents and antibodies

The pharmacological inhibitors BIX02189 and WNK463 were obtained from Selleckchem (Houston, TX, USA) and trametinib from LC Laboratories (Woburn, MA, USA). General chemical reagents were purchased from Sigma–Aldrich (St Louis, MO, USA), Merck (Darmstadt, Germany) or BD Biosciences (San Jose, CA, USA).

The antibodies were obtained as follows: anti‐MEK5 was from Enzo Life Sciences (Farmingdale, NY, USA); anti‐GAPDH and anti‐ERK1/2 from Santa Cruz Biotechnology (Santa Cruz, CA, USA); anti‐pERK1/2, anti‐MEK1/2, anti‐pMEK1/2, anti‐WNK1 and anti‐MEKK2 from Cell Signaling Technologies (Danvers, MA, USA); anti‐Calnexin from Stressgen Bioreagents (Victoria, BC, Canada); anti‐Ki‐67 (clone SP6) from Vitro Master Diagnostica (Granada, Spain); anti‐pWNK1 from R&D Systems (Minneapolis, MN, USA); and horseradish peroxidase‐conjugated secondary antibodies were from Bio‐Rad Laboratories (Hercules, CA, USA). Antibodies raised against ERK5, pERK5 or pMEK5 were developed in our laboratory and have been described.^19^, ^25^, ^26^ The pERK1/2 antibody conjugated to PE fluorophore was obtained from Biolegend (San Diego, CA, USA) and the anti‐pERK5 conjugated to AF488 from Santa Cruz Biotechnology (p‐ERK 5 Antibody (1.T218/Y220) Alexa Fluor^®^488, sc‐135760 AF488).

### 2.2 Cell culture and lentiviral infection

SKOV3 cell line was obtained from the ATCC. OVCAR8, A2780 and IGROV1 ovarian cancer cell lines were from Pharmamar (León, Spain). Their authenticity was checked by STR profiling at the University Hospital of Salamanca. All ovarian cancer cell lines were cultured at 37°C in a humidified atmosphere in the presence of 5% CO_2_ and 95% air. Cells were grown in DMEM medium containing high glucose (4.5 g/L), l‐glutamine 4 mM and l‐pyruvate 5 mM (SKOV3 and OVCAR8) or RPMI medium containing l‐glutamine 4 mM (A2780 and IGROV1). The medium was supplemented with 10% FBS and antibiotics (penicillin 100 U/mL and streptomycin 100 μg/mL). Cell culture media, FBS and penicillin–streptomycin were purchased from GIBCO BRL (Gaithersburg, MD, USA). Cells were maintained in culture for up to 4 months and mycoplasma testing was periodically performed.

Knockdown of WNK1, MEKK2, MEK5 and ERK5 were performed as described^27^ by infection of cell lines with TRC lentiviral pLKO vectors containing human *WNK1* (gene set RHS4533‐EG65125), *MAP3K2* (gene set RHS4533‐EG10746), *MAP2K5* (gene set RHS4533‐EG5607) or *MAPK7* (gene set RHS4533‐EG5598) sequences purchased from Thermo Fisher Scientific (Waltham, MA, USA).

### 2.3 Immunoprecipitation, Western blotting and phosphokinase array

Cells were washed twice with phosphate‐buffered saline and lysed with ice‐cold lysis buffer (140 mM NaCl, 50 mM EDTA, 10% glycerol, 1% Nonidet P‐40, 20 mM Tris–HCl, pH 7.0, 1 mM PMSF, 1 mM Na_3_O_4_V, 10 μM pepstatin, 10 μg/mL aprotinin, 10 μg/mL leupeptin, 25 mM β‐glycerolphosphate, 10 mM NaF). Cell lysates were then centrifuged at 4°C /17,000×*g*/10 min, the supernatants transferred to new tubes and protein concentration measured by the BCA Assay (Thermo Fisher Scientific). Immunoprecipitation and Western blotting were carried out as described.^28^

For the Human Phospho‐Kinase Array (ref. ARY003B, R&D Systems), cell lysates of the ovarian cancer cell lines were hybridised to the array following provider's instructions for simultaneously detecting the relative levels of kinase phosphorylation sites. After chemiluminescent detection, the quantitation of the pixel intensity of each antibody spot was analysed with the Image Studio Lite V5.2 software (LI‐COR Biosciences, Lincoln, NE, USA) and represented according to the pixel intensity with Microsoft Excel (Microsoft Corporation, Redmond, WA, USA).

### 2.4 Patients

Ovarian cancer samples (*n* = 63) were obtained from 57 patients diagnosed with high‐grade (*n* = 35 samples) and low‐grade (*n* = 8 samples) ovarian serous carcinoma (OSC), serous borderline tumour (SBT) (*n* = 13 samples) and endometrioid carcinoma (*n* = 7 samples) at the University Hospital of Salamanca (Table 1), following the Declaration of Helsinki on ethical principles for medical research involving human subjects. Patients provided written informed consent for the usage of the samples and their respective clinical data which were approved by the Institutional Review Board Ethics Committee on Human Research of the hospital. Frozen human ovarian samples were selected by H&E staining to contain at least 90% of tumoural cells. The day of the experiment, samples were washed twice with PBS to remove blood. Samples were then homogenised at a ratio of 1.6 mL of ice‐cold lysis buffer supplemented with 1% Triton X‐100 per 100 mg of tissue using a Dispomix apparatus (L&M Biotech, Holly Springs, USA). After centrifugation at 4°C/17,000×*g*/20 min, the supernatants were transferred to new tubes and protein concentration was measured by the BCA assay. Quantitative measurements of Western blot bands density were carried out using the Image Lab Software 6.0 (Bio‐Rad Laboratories). Densitometric normalisation of ovarian cancer samples was performed using the signal of the ovarian normal sample as internal control among different blots. OVCAR8 and H460 cell extracts were also used as additional internal controls. Autoradiograms that yielded very similar signals of internal controls were selected to quantitate pWNK1 and pERK1/2. Box‐plot representations and Kaplan–Meier survival curves of ovarian cancer patients were generated using the GraphpadPrism 5.0 software (La Jolla, CA, USA). The threshold for including patients into the low/high expression cohorts was set at 10 a.u. for pWNK1 and 4 a.u. for pERK1/2. Survival curve analyses were performed on those patients with available survival data (*n* = 52).

**TABLE 1:** Characteristics of patients from the University Hospital of Salamanca (Spain)

TBN	Age	Ovary	Histology	Grade	Stage	Size (weight)
2124	31	LEFT	OSC	High	N.A.	10 cm
2181	39	LEFT+**RIGHT**	OSC	High	III	Right: 11 cm (276 g); Left: 5 cm (72 g)
2182	39	**LEFT**+RIGHT	OSC	High	III	Right: 11 cm (276 g); Left: 5 cm (72 g)
3044	41	LEFT+RIGHT	OSC	High	I	Right: 10 cm (206 g); Left: 15 cm (518 g)
848	45	RIGHT	OSC	High	N.A.	206 g
3389	46	LEFT+RIGHT	OSC	High	N.A.	Right: 5 cm; Left: 6 cm
3192	48	LEFT+RIGHT	OSC	High	III	Right: 8 cm (210 g); Left: 9 cm (229 g)
5415	48	LEFT+RIGHT	OSC	High	I	Right: 9 cm; Left: 13 cm
439	49	N.A.	OSC	High	N.A.	18 cm (546 g)
1964	49	LEFT+**RIGHT**	OSC	High	N.A.	Right: 4 cm; Left: 5 cm
3886	49	LEFT+RIGHT	OSC	High	III	Right: 5 cm (26 g); Left: 6 cm (56 g)
5511	51	RIGHT	OSC	High	N.A.	10 cm
1552	54	**LEFT**+RIGHT	OSC	High	III	Right: 13 cm; Left: 11 cm
2319	54	LEFT+RIGHT	OSC	High	I	Right: 7 cm (105 g); Left:4 cm
261	55	LEFT+RIGHT	OSC	High	III	Right: 8.5 cm (135 g); Left: 8.5 cm (150 g)
1515	56	LEFT+RIGHT	OSC	High	N.A.	Right + Left: 6.5 cm
3049	56	LEFT+RIGHT	OSC	High	III	Right: 11 cm; Left: 8 cm
388	57	RIGHT	OSC	High	N.A.	14 cm (430 g)
615	57	LEFT+RIGHT	OSC	High	III	Right: 12 cm (872 g); Left: 2 cm
122	62	RIGHT	OSC	High	N.A.	N.A.
1808	63	LEFT	OSC	High	N.A.	4.5 cm
105	66	**LEFT**+RIGHT	OSC	High	N.A.	Right: 6 cm; Left: 12 cm (260 g)
106	66	LEFT+**RIGHT**	OSC	High	N.A.	Right: 6 cm; Left: 12 cm (260 g)
1082	66	LEFT	OSC	High	III	7.5 cm (92 g)
2702	67	LEFT	OSC	High	N.A.	9 cm
1189	74	LEFT+RIGHT	OSC	High	III	Right: 14 cm; Left: 1.5 cm
2136	74	LEFT+RIGHT	OSC	High	N.A.	Right: 12 cm (450 g); Left: 497 g
3148	74	LEFT+RIGHT	OSC	High	N.A.	Right: 4 cm; Left: 5.5 cm
5251	75	LEFT	OSC	High	III	5 cm
1389	76	RIGHT	OSC	High	N.A.	8 cm
1092	77	N.A.	OSC	High	N.A.	N.A.
2174	77	RIGHT	OSC	High	III	12 cm (495 g)
1440	82	LEFT+**RIGHT**	OSC	High	N.A.	Right: 6 cm (90 g) Left: 1.2 cm
2622	83	LEFT	OSC	High	II	19 cm (2150 g)
1469	93	LEFT+RIGHT	OSC	High	N.A.	Right + Left: 4.5 cm
997	42	LEFT	OSC	Low	N.A.	21 cm
2139	48	LEFT+RIGHT	OSC	Low	N.A.	Right: 2.5 cm; Left: 4 cm
640	53	LEFT+**RIGHT**	OSC	Low	III	Right: 21 cm; Left: 17 cm (1504 g)
641	53	**LEFT**+RIGHT	OSC	Low	III	Right: 21 cm; Left: 17 cm (1504 g)
1555	57	**LEFT**+RIGHT	OSC	Low	III	Right: 9 cm; Left: 18 cm (1950 g)
2533	60	LEFT+RIGHT	OSC	Low	II	Right: 15 cm; Left: 6 cm
950	66	**LEFT**+RIGHT	OSC	Low	III	Right: 20 cm; Left: 12 cm (200 g)
951	66	LEFT+**RIGHT**	OSC	Low	III	Right: 20 cm; Left: 12 cm (200 g)
1075	19	LEFT+RIGHT	SBT		I	Right: 13 cm (732 g); Left: 1.5 cm
2193	25	LEFT+**RIGHT**	SBT		II	Right: 15 cm (1240 g); Left: 6 cm
2194	25	**LEFT**+RIGHT	SBT		II	Right: 15 cm (1240 g); Left: 6 cm
5494	29	LEFT+RIGHT	SBT		N.A.	Right: 12 cm; Left: 13 cm
2656	34	LEFT+**RIGHT**	SBT		N.A.	Right: 12 cm (200 g); Left: 7 cm (144 g)
2657	34	**LEFT**+RIGHT	SBT		N.A.	Right: 12 cm (200 g); Left: 7 cm (144 g)
1655	37	RIGHT	SBT		N.A.	6 cm
2460	46	LEFT	SBT		N.A.	9.5 cm (398 g)
5342	52	RIGHT	SBT		I	12 cm
5441	52	LEFT+**RIGHT**	SBT		N.A.	Right: 20 cm; Left: 13 cm
5317	59	LEFT+**RIGHT**	SBT		I	Right: 13 cm; Left: 15 cm
1019	61	**LEFT**+RIGHT	SBT		I	Right: 4.5 cm (25 g); Left: 10 cm (320 g)
695	78	N.A.	SBT		N.A.	8 cm
272	47	**LEFT**+RIGHT	EC	High	III	Right: N.A.; Left: 16 cm
2850	47	RIGHT	EC	High	I	N.A.
610	76	N.A.	EC	High	N.A.	N.A.
5680	39	LEFT	EC	Low	N.A.	12 cm (570 g)
1079	48	RIGHT	EC	Low	III	18 cm (886 g)
1025	55	LEFT	EC	Low	I	10 cm
108	66	RIGHT	EC	Low	I	12 cm

Abbreviations: EC, endometrioid carcinoma; N.A., not available; OSC, ovarian serous carcinoma; SBT, serous borderline tumour; TBN, tumour bank number, correlative numbers in red correspond to tumour samples from the same patient.

### 2.5 In silico studies

The analysis of the most prevalent somatic focal copy number gain events in ovarian cancer patients was performed on the Firebrowse bioinformatic tool (http://www.firebrowse.org). Specifically, source data were obtained from the Broad Institute TCGA Genome Data Analysis Center (2016): Analysis Overview for Ovarian Serous Cystadenocarcinoma (Primary solid tumour cohort) – Broad Institute of MIT and Harvard.^29^ The copy number alteration (CNA) events were identified by GISTIC and classified as deletion (log2(CN ratio) ⇐ −1), loss (−1 < log2(CN ratio) < −0.42), gain (0.58 < log2(CN ratio) < 1.3) or amplification (log2(CN ratio) > = 1.3).

The analyses of CNAs and mRNA levels of *WNK1* as well as their correlation in OSC patients were carried out using the cBioPortal genomic database (http://cbioportal.org) and their available bioinformatic tools.^30^, ^31^ Specifically, the dataset selected for analyses was the Ovarian Serous Cystoadenocarcinoma study from the TCGA (Firehose Legacy, *n* = 606 patient samples). Putative copy‐number calls (*n* = 579) were determined using GISTIC 2.0. Values: −2 = homozygous deletion; −1 = hemizygous deletion; 0 = neutral/no change; 1 = gain; 2 = high level amplification. mRNA expression levels were determined by RNA Seq V2 RSEM (mRNA expression *z*‐scores relative to diploid samples) in those samples from the same TCGA dataset with RNA Seq available data (*n* = 304). To evaluate the impact of WNK1 mRNA levels on the outcome of OSC patients with available survival data (*n* = 303), they were stratified into two groups: the altered group comprises those patients with WNK1 mRNA levels > 2 and the unaltered group those without WNK1 mRNA levels > 2. The survival analysis was also performed on the same dataset by determining mRNA expression levels by Agilent microarray (*n* = 533).

The impact of *WNK1* mRNA expression on the overall survival (OS) of ovarian carcinoma patients was also analysed by RNA‐seq through the Kaplan–Meier plotter database^32^ (http://www.kmplot.com). In this study, patients (*n* = 373) were split into low or high *WNK1* expression cohorts by selecting the best cut off value tool. By using this tool, the database computes all possible cut‐off values between the lower and upper quartiles, and the best performing threshold is automatically selected as threshold. The Kaplan–Meier plotter database was also used to evaluate post progression survival (PPS) (*n* = 735) and progression‐free survival (PFS) (*n* = 1104) of OSC patients.^33^ For these studies, *WNK1* mRNA expression was evaluated by selecting the best scored *WNK1* probe (Affymetrix probe id 211994_at) on the most recent version of the database (2017 version), and patients were split into low or high expression cohorts with the best cut off value tool. The datasets containing the samples for our survival analysis were GSE14764, GSE15622, GSE26193, GSE26712, GSE30161, GSE32062, GSE51373, GSE63885, GSE65986, GSE9891 and TCGA. The array quality controls excluded biased arrays. Cox regression was used to compute the hazard ratio and *p* value. False discovery rates are indicated.

### 2.6 CRISPR assays, xenograft models and immunohistochemical analysis

MEK5 expression was knocked out in OVCAR8 cells using a *MAP2K5* human gene CRISPR/Cas9 knockout kit (reference KN203171) from Origene (Rockville, MD, USA) following manufacturer's instructions. MEK5 levels were tested by Western blotting for the identification of positive knockout clones, whose proliferation rate was then compared with scramble control cells by an MTT assay.

For the in vivo studies, 18 female BALB/c nude mice (Charles River, Wilmington, MA, USA) were divided into three groups of six mice with a similar mean weight. Mice were subcutaneously xenografted with 1 × 10^6^ OVCAR8 scramble, #16 or #19 MEK5 CRISPR cells following standard procedures into both flanks (*n* = 12 tumours per group). Tumours were allowed to engraft for 1 week and measured once a week using a digital caliper until the end point of the experiment. Tumour volume (mm^3^) was calculated by the formula: *V* = *L* × *W* ^2^ × 0.5. All animals were manipulated and sacrificed by authorised personnel at the animal facility following legal and institutional guidelines.

Resected tumours were divided into halves. One of them was immediately frozen in liquid nitrogen and afterwards homogenised and processed for biochemical analyses as previously described.^26^ The other half was formalin fixed, paraffin embedded and sectioned for subsequent immunohistochemical staining with the Ki‐67 antibody using the Bond Polymer Refine Detection kit (Leica Biosystems, Newcastle, United Kingdom).

For the in vivo pharmacological experiment, the group of mice xenografted with scramble cells (Sc) as well as the MEK5 KO mice were further divided into two subgroups. The start point of the experiment was established when the mean tumour volume of each subgroup reached approximately 500 mm^3^. For each condition, one of the subgroups received 100 μL of trametinib (0.5 mg/kg dissolved in 30% PEG, 5% Tween‐80 and 2% DMSO) while the other one received vehicle. Treatments were administered i.p. daily for 5 weeks. Tumour progression of Sc and CRISPR groups were measured weekly. For the analysis of treatment efficacy, the tumour volumes of the trametinib‐treated mice were relativised to their corresponding vehicle‐treated groups.

### 2.7 Cell proliferation

Cells were plated in 24‐well plates at 15,000 (SKOV3, IGROV1 and OVCAR8) or 10,000 (A2780) cells per well (triplicates or quadruplicates), in medium containing or not the appropriate drugs and incubated for the indicated periods of time. Cell proliferation was analysed by an MTT‐based assay as previously described.^28^ Alternatively, cells were plated in 6‐well plates at 100,000 (SKOV3 and IGROV1), 60,000 (OVCAR8) or 50,000 (A2780) cells per well (duplicates or triplicates) and proliferation measured by cell counting using a Z1 Coulter Particle Counter (Beckman‐Coulter, Brea, CA, USA).^34^ Results were normalised to control and presented as the mean ± SD of an experiment that was repeated two or three times.

### 2.8 In vitro kinase assay

In vitro kinase assay was performed on cell lysates of OVCAR8 cells previously treated with the indicated doses of trametinib for 2 h. After immunoprecipitation of 1 mg of protein with the ERK5 antibody, samples were washed twice with lysis buffer and then twice with kinase buffer (20 mM Hepes pH 7.6, 20 mM MgCl_2_, 25 mM β‐glycerolphosphate and 1 mM Na_3_O_4_V in MiliQ water). After that, 40 μL of kinase buffer containing 0.16 μM ATP was added to each sample, which were then incubated for 30 min/30°C in a water bath. Finally, the reaction was stopped by adding 25 μL of 4× sample buffer to each sample, which were subsequently analysed by Western blotting.

### 2.9 Human plasma‐derived 3D (HuP3D) cultures of primary ovarian tumour biopsies

Human OSC patient samples and matched plasma samples were obtained from the Sanford Biobank (Sioux Falls, SD, USA) (Table 2). Informed consent was obtained from all subjects with approval from the Sanford Health Institutional Review Board and in accordance with the Declaration of Helsinki. Tumour biopsies were harvested to collect tumour cells using a commercially available tumour dissociation kit (Miltenyi Biotec, Bergisch Gladbach, Germany), as per manufacturer's instructions. Primary ovarian isolated cells were embedded in a human plasma‐derived 3D culture (HuP3D) made with the plasma from the same ovarian cancer patient. HuP3D cultures were engineered through the cross‐linking of the corresponding patient's fibrinogen into fibrin as previously described.^35^ Briefly, plasma was first mixed with tumour cell suspension (to yield 3 × 10^4^ cells/per scaffold) prepared in DMEM complete media followed by encapsulation into matrices that were established using calcium chloride (CaCl_2_) as a cross‐linker and trans‐4‐(aminomethyl) cyclohexanecarboxylic acid as a stabiliser, in a 4:4:1:1 ratio, respectively.

**TABLE 2:** Characteristics of patients from Sanford Biobank (SD, USA)

Exp ID	Age	Ovary	Histological type
1	61	Right	OSC
2	63	Left	OSC
3	63	Right	OSC
4	53	Left	N.A.

OSC, ovarian serous carcinoma.

N.A., not available.

HuP3D cultures were allowed to stabilise and at the end of Day 0, primary ovarian tumour cells seeded within the HuP3D matrix were subjected to DMSO control treatment; trametinib (0.25 μM) treatment for 72 h, where trametinib was refreshed after the initial 24 h; BIX02189 (25 μM) treatment from 24 to 72 h, or initial 24 h trametinib treatment followed by an additional 48‐h treatment with a combined dose of trametinib (0.25 μM) and BIX02189 (25 μM). After 72 h, HuP3D cultures were enzymatically digested with collagenase (20 mg/ml for 2−3 hours at 37°C) and cells were retrieved as previously described.^35^, ^36^ Cells retrieved from the digested HuP3D cultures were probed with anti‐EpCAM antibody conjugated to PECy7 fluorophore (Biolegend) to help selective identification of the epithelial‐tumour cells, with anti‐Fibroblast Activation Protein (FAP) antibody conjugated to AF700 fluorophore (R&D Systems) and anti‐CD45 antibody conjugated to BV510 fluorophore (Biolegend) to help remove stromal and immune cells, respectively. Cell viability was evaluated by using a Live/Dead Blue cell stain (Thermo Fischer Scientific).

The commercially available cell stimulation cocktail utilised to establish the positive (activated) control for the subsequent determination of pERK1/2 and pERK5 levels was purchased from Invitrogen (Waltham, MA, USA). Retrieved cells were activated with this cocktail for at least 6 h as per manufacturer's recommendations. Data acquisition was completed by collecting information for a specified number of events determined by counting beads. For all analyses, a minimum of 10,000 events were acquired using BD FACS Fortessa and FACSDiva v6.1.2 software. The EpCAM+ expressing ovarian tumour cell counts were always normalised to a predetermined number of counting beads (Biolegend) and mean fluorescence intensity (MFI) ratios for each of the targets (pERK1/ and pERK5) were assessed with respect to the corresponding fluorescence minus one (FMO) control in the EpCAM+ cells after fixation/permeabilisation treatment. The data were analysed using FlowJo program v10 (Ashland, OR, USA).

### 2.10 Statistical analyses

Fisher's exact test was used for the analyses of variance between variables and comparison of continuous variables between groups were carried out using a two‐sided Student's *t*‐test in the in vitro studies. Kolmogorov–Smirnov test was used to analyse whether data of the in vivo tumour growth experiments followed normal distribution, and one‐way ANOVA was used to compare tumour growth between the different treatment groups. The association of pWNK1 expression or pERK1/2 expression with the outcome of patients from the University Hospital of Salamanca was evaluated by Kaplan–Meier survival curves performed with the GraphpadPrism 5.0 software according to the Gehan–Breslow–Wilcoxon Test and providing information about hazard ratios and confidence intervals. The association of a specific variables with outcome in relation to other variables was performed using a multivariable Cox regression model that included the most relevant clinical variables. The differences between groups were considered significant at *p* value ≤ .05 (*), *p* ≤ .01 (**) or *p* ≤ .001 (***). General statistical analyses were performed using the SPSS 19.0 software (SPSS Inc., Chicago, IL, USA). Additional information is described in the appropriate figure legends.

## 3 RESULTS

### 3.1 Active kinase profiling of ovarian cancer cells

**FIGURE 1:**
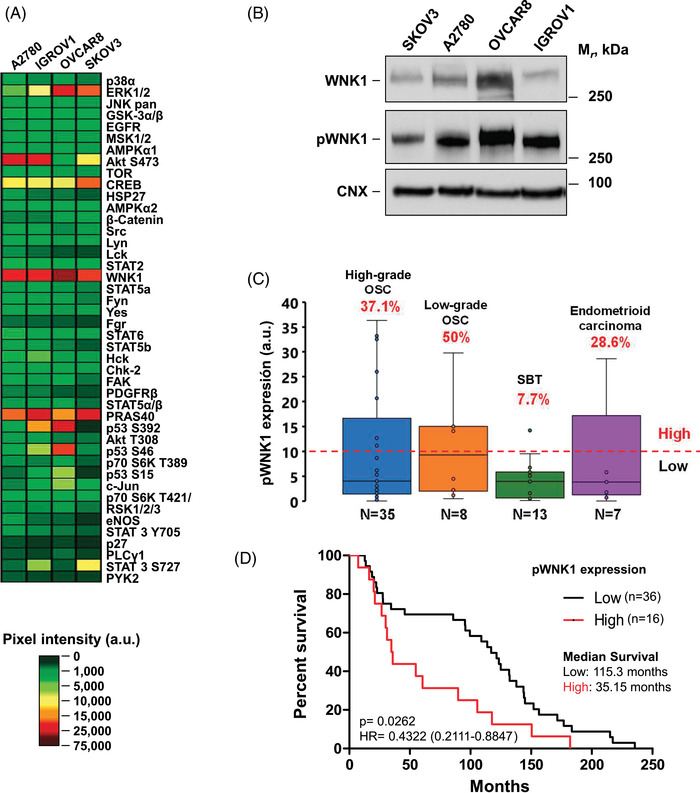
Identification of WNK1 as an active kinase in ovarian cancer. Impact on patient survival. (A) 300 μg of cell lysate from the four ovarian cancer cell lines were hybridised to the phosphokinase array following manufacturer's instructions. The immunodetected signals corresponding to the amount of phosphorylated protein bound were quantified and represented as indicated in Methods section. (B) 70 μg of whole‐cell lysates were used to detect WNK1 and pWNK1 expression in the four cell lines by Western blotting. Calnexin was used as loading control. (C) Box plots representing the levels of pWNK1 in 63 epithelial ovarian cancer samples obtained from 57 patients from the University Hospital of Salamanca, which were quantified from Western blot analysis (shown in Figure S1) using the ImageJ software. The red line represents the threshold value (10 a.u.) selected for sorting the patients into the high or low pWNK1 expression groups. The number of samples from each subtype and the percentage belonging to the high expression cohort are shown. OSC, ovarian serous carcinoma; SBT, serous borderline tumour. (D) Kaplan–Meier curve of the above patients with available clinical data (*n* = 52), comparing the overall survival of those expressing high levels of pWNK1 (*n* = 16, red line) with those expressing low levels (*n* = 36, black line). The median overall survival of each cohort and *p* value are shown.

In attempting to uncover novel pathophysiological players in ovarian cancer, we analysed the activation status of proteins that participate in several signalling routes. A total of 43 kinase phosphorylation sites were analysed in four different epithelial ovarian cancer cell lines, OVCAR8, SKOV3, IGROV1 and A2780,^37^ as epithelial tumours are the most frequent type of ovarian cancer. Antibody arrays were used to explore the phosphorylation status of those proteins, considering that such posttranslational modification is regularly used as readout of the activity of the distinct signalling pathways in which they participate. These studies showed that the strongest phosphorylated protein across the four ovarian cancer cell lines was WNK1 (Figure 1A). Western blotting analyses confirmed the expression of pWNK1 and WNK1 in the four ovarian cancer cell lines (Figure 1B). In addition, phosphorylation of PRAS40, AKT, ERK1/2 or CREB was also observed.

The levels of WNK1 and pWNK1 were also analysed in a cohort of 57 patients with ovarian cancer (Figure S1). Tumours (*n* = 63, as samples from both ovaries were analysed in six of the 57 patients) were obtained from the Salamanca University Hospital and their pathologic characteristics are included in Table 1. Most of the samples belonged to the OSC histological type, which is the most frequent one.^38^ The immunoblots not only demonstrated distinct patterns of pWNK1 between histological types and tumour grades, but also varying levels of pWNK1 among patients from the same subtype. Thus, quantitation of pWNK1 levels revealed a high activation of this protein in 37.1% of high‐grade OSC (*n* = 35), 50% of low‐grade OSC (*n* = 8), 7.7% of SBT (*n* = 13) and 28.6% of endometrioid carcinomas (*n* = 7) (Figure 1C).

### 3.2 WNK1 and pWNK1 expression linked to patient outcome in ovarian cancer

A potential relationship between WNK1 activation, molecular alterations or expression and patient outcome was analysed. Since data on WNK1 activation were not available in public databases, we used clinical data from the patient cohort of our University Hospital and from which pWNK1 levels were obtained. Kaplan–Meier analyses demonstrated that a higher expression of pWNK1 negatively impacted on survival (Figure 1D), revealing a 3.28‐fold decrease in the median survival in high pWNK1 patients (35.15 months) compared with those presenting low levels (115.3 months). Multivariate analysis confirmed association between pWNK1 expression and detrimental survival (Table S1)

**FIGURE 2:**
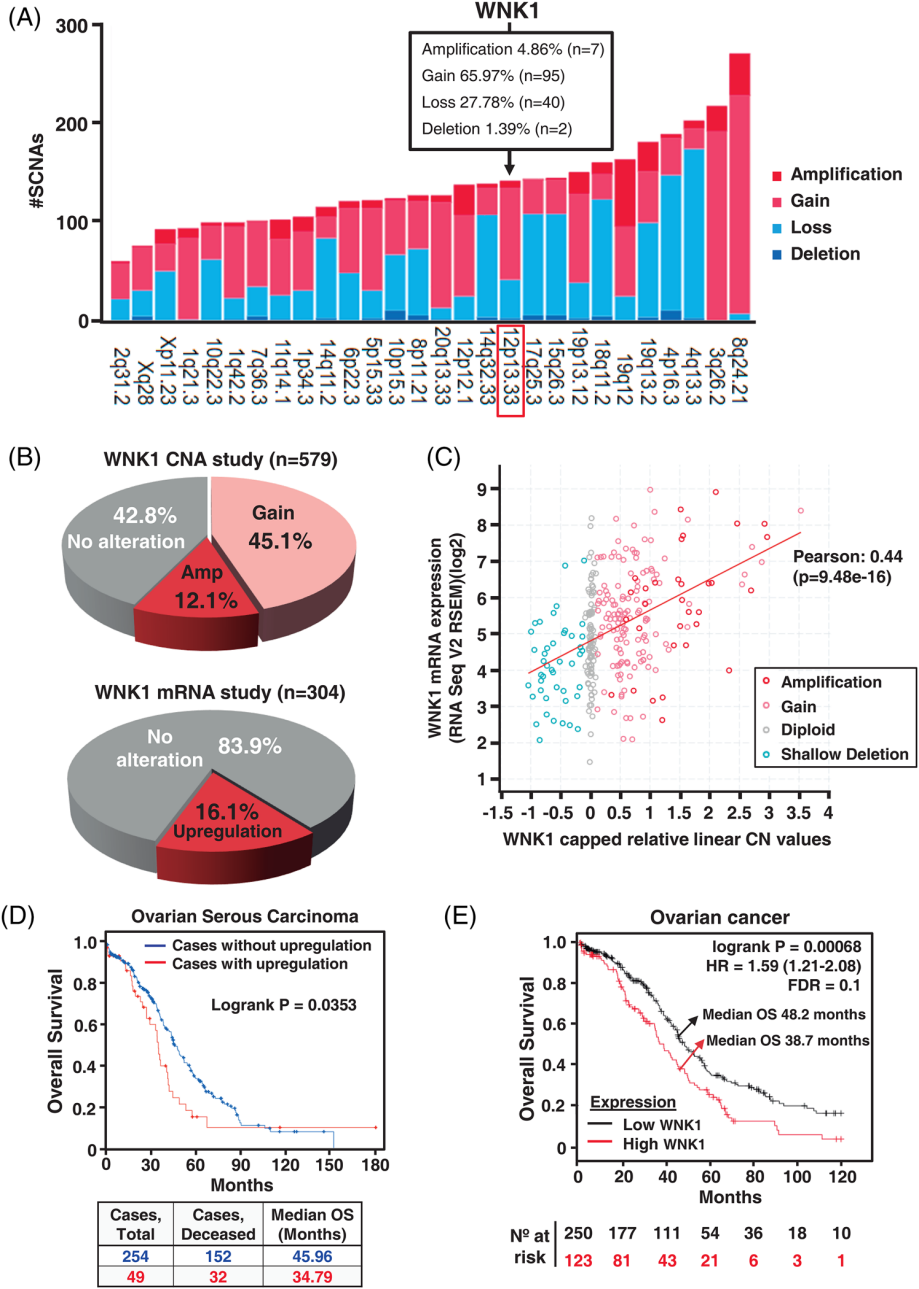
Genomic analysis of *WNK1* and relevance of its dysregulation in ovarian cancer patient's outcome. (A) Firebrowse bioinformatic tool was used for the identification of differentially expressed marker focal events in the ovarian cancer cystadenocarcinoma dataset (*n* = 579 samples), using Student's *t*‐test. The top 28 focal level copy number alterations were ranked and represented from lowest to highest frequency. (B) The cBioPortal database was explored to determine gains, high‐level amplifications as well as upregulated mRNA levels of *WNK1* gene in ovarian cancer patients. The data were collected, quantified and represented as percentage from the total patients with available data included in the study, as indicated in Methods section. (C) Relationship between *WNK1* copy number alterations (CNA) and *WNK1* mRNA expression of ovarian cancer samples collected in the TCGA Firehose Legacy dataset. The Pearson correlation coefficient between these variables and the tendency line (red line) are shown. The copy number datasets were generated by the GISTIC algorithm. (D) Overall survival curves of those ovarian serous carcinoma patients from the TCGA Firehose Legacy dataset with available RNAseq mRNA expression and clinical data (*n* = 303). Patients harbouring *WNK1* mRNA upregulated levels (red line) were compared to those without such alteration (blue line). The *p* value of the study, follow‐up (months) and median overall survival of each cohort are indicated. (E) 120 months follow‐up Kaplan–Meier analysis of the relationship between *WNK1* mRNA expression levels and overall survival in ovarian cancer patients (*n* = 373) collected in the RNA‐seq section of the Kaplan–Meier plotter database. The *p* value, hazard ratio, median overall survival and number of patients at risk in the low and high expression groups are indicated; FDR = 0.20.

To evaluate the somatic CNAs most frequently present in ovarian cancer, we used the Firebrowse online tool.^39^ Analysis of the Ovarian Serous Cystadenocarcinoma dataset (TCGA, *n* = 579) revealed that chromosomal cytoband 12p13.33, which comprises the *WNK1* gene, was among the top‐ranked altered genomic locations (Figure 2A), reaching statistical significance (*p* = 8.7e−07). Analysis of the Ovarian Serous Cystadenocarcinoma dataset (TCGA, Firehose Legacy) using the cBioportal genomic database^40^ showed the presence of *WNK1* gene amplifications (12.1%) or gains (45.1%) in the ovarian tumours (*n* = 579) (Figure 2B, upper panel), as well as *WNK1* mRNA upregulation in 16.1% of the cases (*n* = 304) (Figure 2B, lower panel). As expected, *WNK1* gene copy number positively and significantly correlated with the *WNK1* mRNA expression level (Pearson correlation coefficient = 0.44, *p* = 9.48e−16) (Figure 2C).

The potential relationship between RNA‐seq quantified *WNK1* mRNA expression levels and patient outcome in those patients with annotated survival data (*n* = 303) was then explored. As shown in Figure 2D, *WNK1* mRNA upregulation significantly associated with worse prognosis (*p* = .0353). Similar results were obtained when the analysis was performed with microarray quantified mRNA levels (*n* = 533, *p* = 4.498e−3) (Figure S2A). Complementary analysis using the Kaplan–Meier Plotter online tool,^41^ which allows investigation of the relationship between the levels of RNA‐seq quantified mRNA expression and patient outcome, showed that high expression of *WNK1* negatively correlated with OS in ovarian cancer patients (*n* = 373, *p* = .00068) (Figure 2E). Moreover, higher *WNK1* expression levels were also associated to worse PPS and PFS (Figures S2B and C, respectively).

### 3.3 Role of the WNK1–MEKK2–MEK5–ERK5 axis in ovarian cancer cell proliferation

**FIGURE 3:**
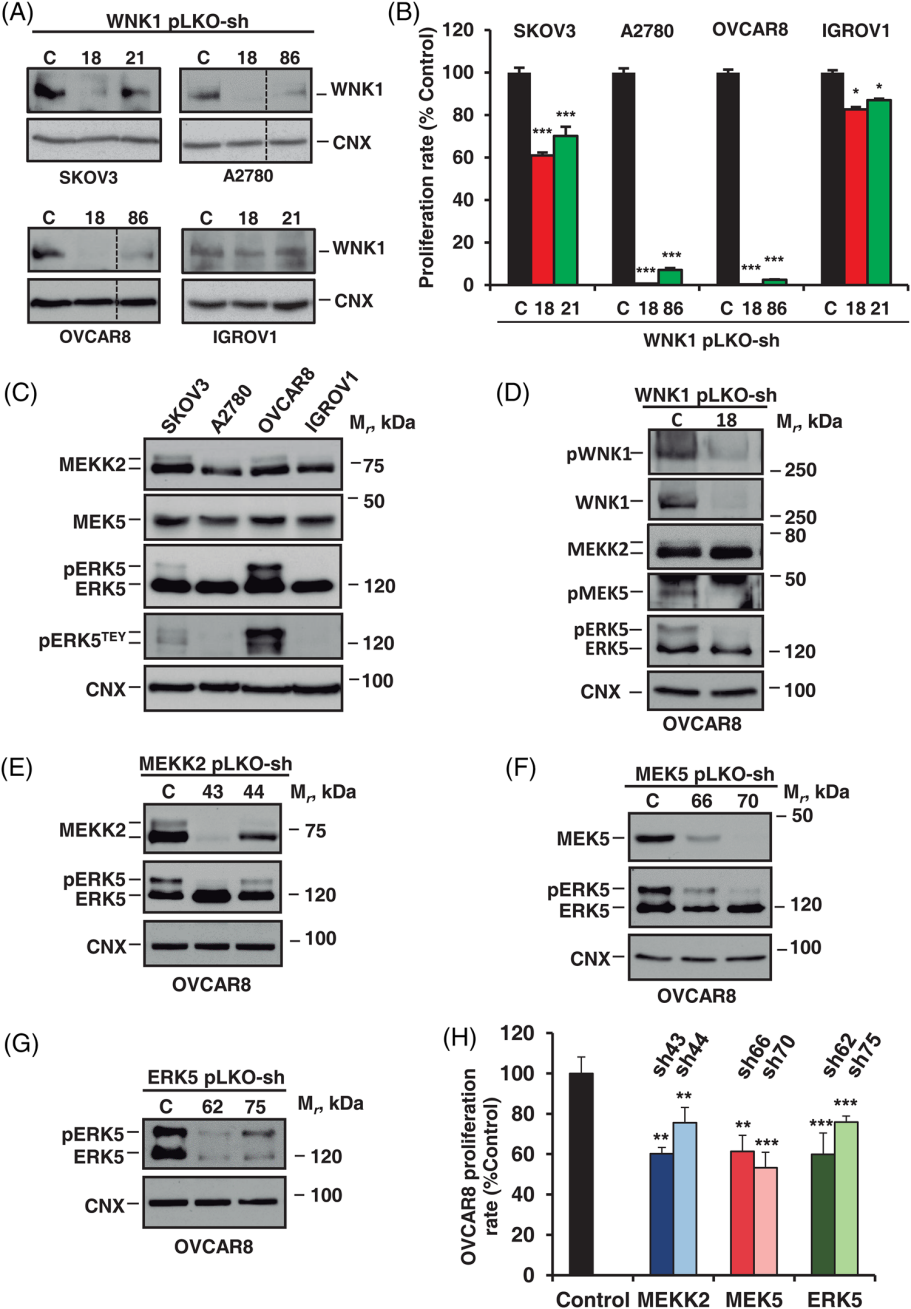
Anti‐proliferative effect of WNK1–ERK5 axis knockdown. (A) Cells were infected with a pLKO shControl sequence or two different WNK1 specific shRNA sequences. 70 μg of cell extracts were used to determine the WNK1 knockdown by Western blotting. Calnexin was used as loading control. Dashed line indicates that lanes were cut out from immunoblots. (B) The effect of WNK1 knockdown (sh18 and sh21 or sh86) on cell proliferation was measured at 3 days of culture by the MTT assay. Data are presented as the mean ± SD of an experiment that was repeated three times. *, *p* ≤ .05; ***, *p* ≤ .001. (C) 70 μg of whole‐cell lysates were used to detect MEKK2 and MEK5 expression in the four ovarian cancer cell lines by Western blotting with their corresponding antibodies. ERK5 activation was analysed by immunoprecipitating 1 mg of protein followed by Western blotting with the anti‐ERK5 or anti‐pERK5 antibodies. Calnexin was used as loading control. (D) OVCAR8 cells infected with pLKO shControl or sh18 were lysed and protein levels were evaluated on 70 micrograms of cell extracts by Western blotting with their corresponding antibodies. ERK5 and pMEK5 were analysed by immunoprecipitating 1 mg of protein followed by Western blotting with anti‐ERK5 or anti‐pMEK5. Calnexin was used as loading control. (E) Knockdown of MEKK2, (F) MEK5 or (G) ERK5 was carried out by lentiviral infection with specific shRNA sequences. MEKK2, MEK5 (70 μg of cell extracts) and ERK5 (1 mg of immunoprecipitated protein) were analysed by Western blotting with the appropriate antibodies. Calnexin was used as loading control. (H) The effect of MEKK2, MEK5 and ERK5 knockdown on cell proliferation was measured at 3 days of culture by the MTT assay. Data are presented as the mean ± SD of an experiment that was repeated three times. **, *p* ≤ .01; ***, *p* ≤ .001.

To analyse the relevance of WNK1 in the control of the proliferation of ovarian cancer cells, knockdown experiments were initially carried out. From five sequences of shRNA, initial experiments allowed selection of sequences #18 and #86 to knockdown WNK1 expression in A2780 and OVCAR8 cells and sequences #18 and #21 to knockdown WNK1 expression in SKOV3 and IGROV1 cells. Knockdown of WNK1 (Figure 3A) was accompanied by a profound inhibitory effect on the proliferation of A2780 and OVCAR8 and moderate in SKOV3 (Figure 3B). The low efficacy of the knockdown of WNK1 in IGROV1 (Figure 3A) was in accordance with the restricted effect on its proliferation (Figure 3B).

All the ovarian cancer cell lines expressed the WNK1 downstream kinases MEKK2, MEK5 and ERK5 (Figure 3C). Western blotting with antibodies directed to phosphorylated ERK5, which are used as readout of ERK5 activation status,^23^ demonstrated that this kinase was particularly active in OVCAR8 cells. In this cell line, the knockdown of WNK1 not only decreased its amount but also decreased pWNK1 levels (Figure 3D). Moreover, the WNK1 knockdown affected MEK5 phosphorylation and consequently decreased the amount of the slower migrating form of ERK5 (pERK5) (Figure 3D). A small decrease in the intensity of the upper band within the MEKK2 blot and a slight increase in the lower band was also observed. MEKK2 or MEK5 knockdown behaved in a similar way with respect to ERK5 activation (Figures 3E and F). Cells in which MEKK2, MEK5 or ERK5 were knocked down (Figures 3E, F and G, respectively) were used to explore the relevance of the route in ovarian cancer proliferation. As observed in Figure 3H, decreasing the levels of each of these kinases negatively impacted on the proliferation of OVCAR8 cells. Together, these results indicate that the WNK1–MEKK2–MEK5 axis is responsible for the constitutive activation of the ERK5 route in these cells. Moreover, the above data suggest that this route plays a relevant role in the proliferation of ovarian cancer cells, as represented by the OVCAR8 model.

**FIGURE 4:**
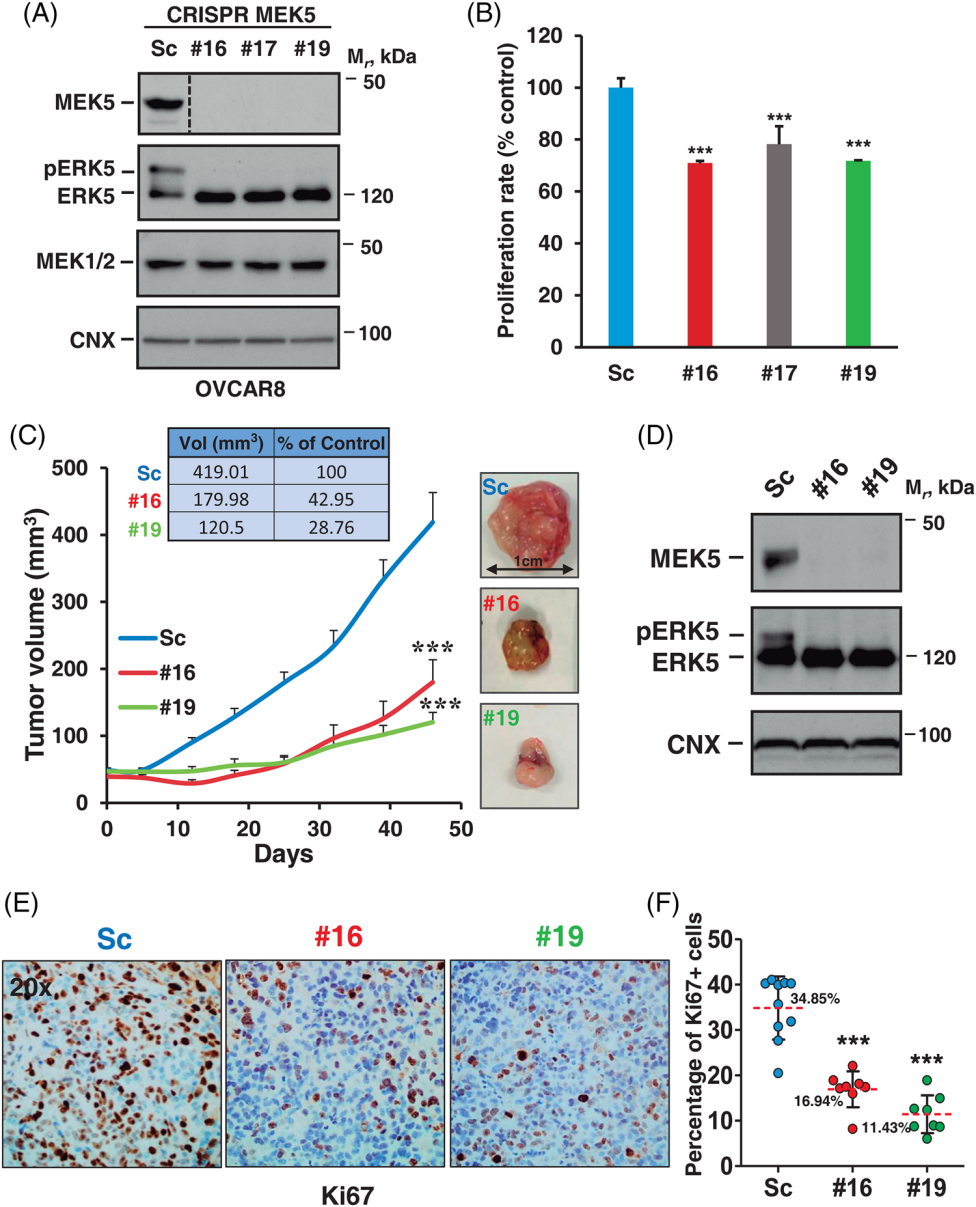
Tumour growth inhibition caused by MEK5 knockout. (A) Western blot showing lack of MEK5 expression in the different OVCAR8 MEK5 CRISPR clones when compared with the control OVCAR8 scramble cells (Sc). ERK5 expression was analysed by immunoprecipitating 1 mg of protein followed by Western blotting with the anti‐ERK5 antibody. MEK1/2 was detected by Western blotting on 70 μg of cell extracts. Calnexin was used as loading control. Note: A lane was cut out from the immunoblot. (B) Effect of MEK5 knockout on the cell proliferation of OVCAR8 cells. Control OVCAR8 Sc cells or OVCAR8 CRISPR MEK5 clones were plated in p6 wells and cultured for 3 days. The proliferation rate was measured by cell counting and represented as percentage from control OVCAR8 Sc cells. Data are presented as the mean ± SD of an experiment that was repeated three times. ***, *p* ≤ .001. (C) Tumour growth evolution of the mice xenoinjected with the OVCAR8 Sc cells or MEK5 CRISPR cells (#16 and #19 clones) (*n* = 12 tumours, per group). The mean tumour volume and SEM error bars of each group are represented. The experiment was stopped when mice tumours from the Sc group reached a volume of 420 mm^3^. The significative *p* values between groups are indicated (***, p ≤ .001). Data in the inset table indicate the volume percentage of MEK5 CRISPR tumours when compared with scramble tumours. Representative pictures of the tumour size of each group are shown. (D) Western blot showing MEK5 and ERK5 expression in tumours collected at the end of the in vivo experiment. (E) Representative Ki67 immunostaining 20x pictures of tumoural tissue from each condition (scramble and MEK5 CRISPR clones). (F) Quantitation of Ki67 immunostaining from 8 to 10 different images of each condition is represented as percentage of Ki67 positive cells with respect to total. The average percentage (dotted red line), ± SD and *p* value (***, *p* ≤ .001) are indicated for each MEK5 CRISPR clone.

To explore the in vivo growth properties of ovarian cancer cells in which the WNK1–MEKK2–MEK5–ERK5 route has been interrupted, we deleted MEK5 of OVCAR8 cells using CRISPR/Cas9. MEK5 knockout was achieved in several clones (Figure 4A) and resulted in disappearance of the slower migrating form of ERK5. Cell proliferation studies showed that the MEK5 knockout clones grew less than OVCAR8 cells transfected with a scramble (Sc) control sequence (Figure 4B). To follow the in vivo tumour generation characteristics of the MEK5 knockout cell lines, we injected cells from two MEK5 knockout clones into the flanks of nude mice and monitored their growth along time. All injected cell lines gave rise to tumours (Figure 4C). However, those derived from MEK5 CRISPR #16 and #19 clones were significantly smaller than the ones created by injecting OVCAR8 Sc cells (*p* = .00028 and *p* = .000021, respectively). Western blotting of dissected tumours confirmed that the levels of MEK5 were undetectable in the samples derived from #16 and #19 clones (Figure 4D). The tumours expressed ERK5, but the phosphorylated form of that protein could only be detected in tumour samples from mice injected with Sc‐OVCAR8. Of note, the decrease in tumour size was related to a lower degree of cell proliferation, as indicated by tumour staining with the proliferation marker Ki‐67 (Figures 4E and F).

### 3.4 The WNK1–ERK5 axis modulates the anti‐tumoural properties of MEK1/2 inhibitors

**FIGURE 5:**
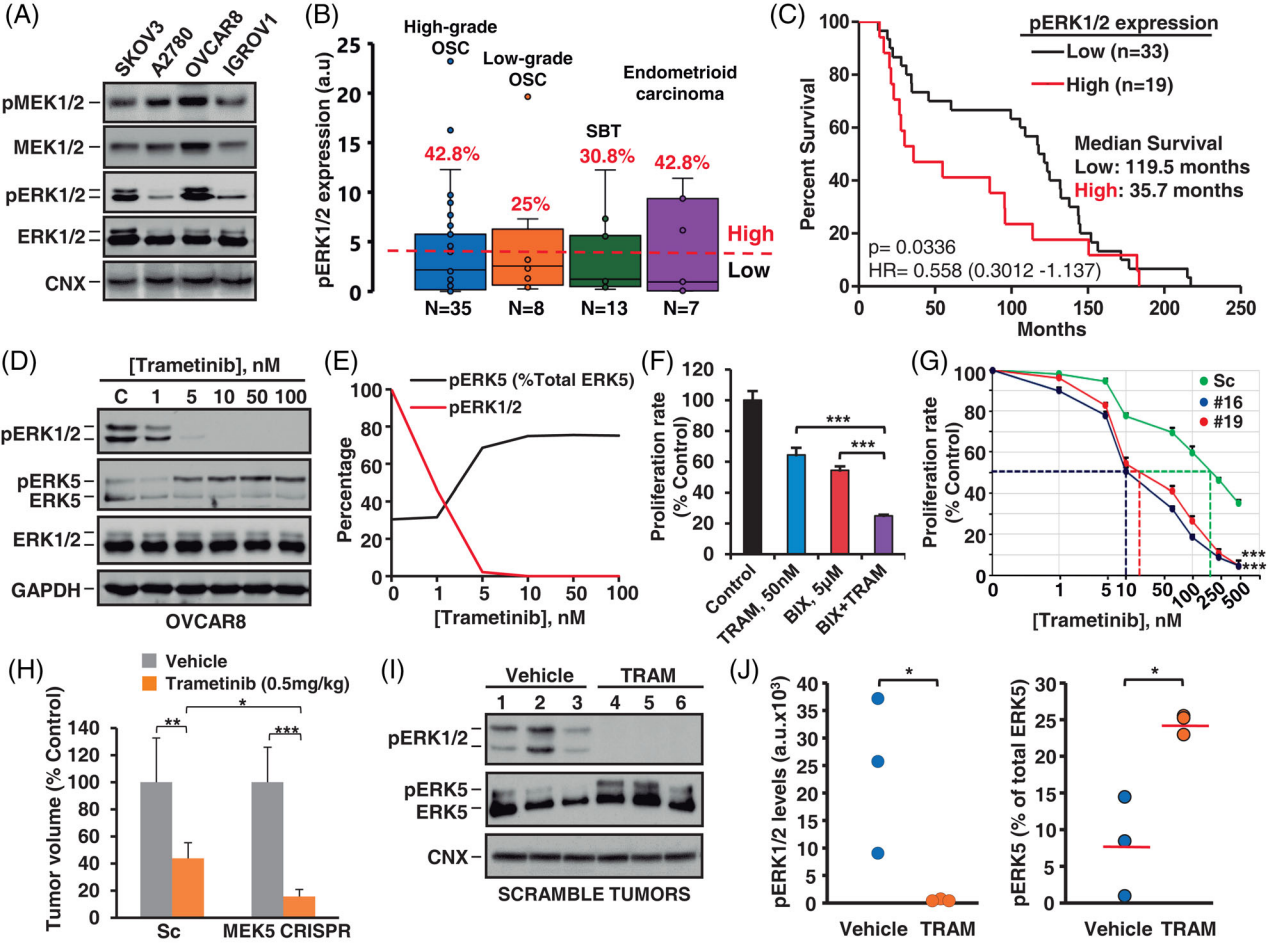
Impact of pERK1/2 on patient survival. Anti‐tumoural efficacy of double pathway (ERK5 and ERK1/2) blockade. (A) MEK1/2, pMEK1/2, ERK1/2 and pERK1/2 expression were determined by Western blotting. (B) Levels of pERK1/2 in 63 epithelial ovarian cancer samples quantified from immunoblots in Figure S1 using the ImageJ software. The red line represents the threshold value (4 a.u.) selected for sorting the patients into the high or low pERK1/2 expression groups. (C) Kaplan–Meier curve of the above patients with available clinical data (*n* = 52), comparing the overall survival of those expressing high levels of pERK1/2 (*n* = 19, red line) with those expressing low levels (*n* = 33, black line). (D) OVCAR8 cells were treated with trametinib for 4 h and pERK1/2, ERK1/2 and ERK5 expression determined by Western blotting. (E) pERK1/2 and pERK5 levels were quantified from the previous immunoblots (in D) by using the Image Lab software and represented as percentage from control untreated cells. (F) OVCAR8 cells were plated in p6 wells and treated with trametinib and BIX02189, individually and combined, for 3 days. Cell proliferation, measured by cell counting, was represented as percentage from OVCAR8 untreated cells. Data are presented as the mean ± SD of an experiment that was repeated three times. ***, *p* ≤ .001. (G) OVCAR8 scramble cells and OVCAR8 MEK5 CRISPR clones (#16 and #19) were plated in 24‐well dishes, and 24 h later treated with the indicated doses of trametinib for 48 h. Cell proliferation was measured by an MTT assay, and each condition was represented as percentage from their respective untreated cells. Results are expressed as mean ± SD of an experiment that was repeated twice. ***, *p* ≤ .001. (H) Mice xenografted with OVCAR8 Sc or MEK5 CRISPR cells were divided into two groups once they reached an initial mean volume of approximately 500 mm^3^. Each of them received 100 μL of trametinib (0.5 mg/kg) or vehicle, administered i.p. daily for 5 weeks. Tumour progression of Sc and CRISPR groups were measured weekly. At the end point of the experiment, the tumour volume of the trametinib treated mice were relativised to their corresponding untreated (vehicle) groups *, *p* ≤ .05; **, *p* ≤ .01; ***, *p* ≤ .001. (I) Scramble tumours from the vehicle and trametinib treated mice were resected and processed as described in the methods section. pERK1/2 and ERK5 were determined by Western blotting. (J) pERK1/2 (left panel) and pERK5 (right panel) levels from the previous immunoblots (in I) were quantified using the Image Lab software. Comparison of pERK1/2 levels in the scramble tumours treated with vehicle or trametinib was represented as arbitrary units. Comparison of pERK5 expression between groups was represented as percentage of total ERK5 (sum of upper and lower bands). The red lines represent the mean expression values for each group. *, *p* ≤ .05.

In addition to WNK1, the data presented in Figure 1A showed activation of the ERK1/2 route, an ERK5 cognate pathway, in the ovarian cancer cell lines. Western blotting analyses confirmed activation of ERK1/2 as indicated by the presence of pERK1/2 (Figure 5A). To analyse whether pERK1/2 expression related to clinical outcome, we used the same patient population from our hospital employed in the WNK1 studies mentioned above. As shown in Figures 5B and S1, pERK1/2 expression varied among different tumoural samples. Kaplan–Meier analyses of the impact of pERK1/2 expression on patient survival reflected a negative association (Figure 5C). The difference in patient outcome between the low and high pERK1/2 expression cohorts reached statistical significance (*p =* .0336, HR = 0.5851 (0.3012–1.137)), demonstrating a 3.34‐fold decrease in the median survival of high pERK1/2 patients (35.7 months) when compared with those presenting low levels (119.5 months). A multivariate analysis using a cox‐regression model was performed to demonstrate the association of pERK1/2 with poor survival (Table S2).

The finding that the activation status of WNK1 and ERK1/2 predicts a poor outcome in ovarian cancer, prompted us to explore their connection in this disease. Treatment of OVCAR8 cells with the MEK1/2 inhibitor trametinib provoked a dose‐dependent (Figures 5D and E) and time sustained (Figure S3A) decrease in the activation status of pERK1/2. Moreover, trametinib provoked a shift in the mobility of ERK5 compatible with its dual phosphorylation and activation (Figures 5D and E). Pre‐treatment with the MEK5 inhibitor BIX02189 prevented the action of trametinib on ERK5 phosphorylation, indicating that the latter drug acted upstream of MEK5 (Figure S3B). Additional western blot analyses confirmed that the activation mechanism already occurs on the upstream kinase effectors WNK1 and MEK5 (Figure S3C). Trametinib was also able to cause phosphorylation of ERK5 in cell lines in which basal levels of pERK5 were weak (SKOV3) or not detected (A2780) (Figure S3D).

To further support these data, in vitro kinase assays were made. In these experiments, ERK5 acts both as the enzyme and substrate due to its autophosphorylation characteristics. ERK5 autophosphorylation causes the appearance of a hyperphosphorylated form, termed as ppERK5.^27^ *In cellula* treatment of OVCAR8 cells with trametinib increased the intensity of hyperphosphorylated ppERK5 (Figure S3E). While trametinib caused an activation of ERK5, the WNK1 and MEK5 inhibitors (WNK463 and BIX02189, respectively) were both able to reduce pERK5 levels, but did not affect pERK1/2 (Figure S3F). Altogether, these data confirm that pharmacological inhibition of MEK1/2 triggers activation of ERK5 through WNK1.

### 3.5 Anti‐tumoural efficacy of double ERK1/2 and WNK1–ERK5 targeting

The fact that the WNK1–ERK5 route was associated with proliferation in ovarian cancer cells raised the possibility that its activation by trametinib could limit the anti‐tumoural effectiveness of such drug. It was therefore postulated that acting simultaneously on both ERK5 and ERK1/2 should result in a higher anti‐tumoural effect than individually targeting the ERK5 and ERK1/2 routes. MTT metabolisation assays demonstrated that either the treatment with MEK5 or MEK1/2 inhibitors reduced the proliferation of OVCAR8 cells in vitro (Figure 5F). However, combination of both had a higher anti‐proliferative effect than the single treatments. Similar results were obtained when combining the WNK1 inhibitor with trametinib (Figure S3G). An analogous behaviour was also observed in A2780 and SKOV3 cells.

As a complement to the pharmacological combination studies, the effect of MEK1/2 inhibition on OVCAR8 cells genetically manipulated for MEK5 loss of function was analysed. MEK5 knockout by CRISPR favoured the efficacy of trametinib (Figure 5G). Thus, the effect of trametinib was more potent in the two clones of OVCAR8 cells in which expression of MEK5 was eliminated (IC_50_ values of 9.87 nM for clone #16 and 14.56 nM for clone #19) when compared with the scramble control cells (IC_50_ value of 178 nM). Similar results were obtained when combining MEK5 knockdown and trametinib treatment (Figure S3H). Whether this higher anti‐tumoural effect of trametinib in cells deficient in ERK5 pathway could also be reproduced in vivo was also investigated. To that end, mice xenografted with Sc or MEK5 knockout OVCAR8 cells were treated with vehicle or trametinib. Trametinib provoked a higher reduction of the growth of tumours that did not express MEK5 when compared with the scramble tumours (Figures 5H and S4). Western blot analyses, performed on tumours removed from mice on the last day of the experiment, indicated that trametinib abolished the phosphorylation of ERK1/2 in both scrambled cells and clones lacking MEK5 (Figures 5I and 5J left and Figure S3I). In contrast, trametinib caused a statistically significant increase in the phosphorylation levels of ERK5 in scrambled cells (compared to vehicle treatment, *p =* .013, Figure 5J right), but not in clones lacking MEK5, since the lack of the upstream activator of ERK5 impeded its activation (Figure S3I).

### 3.6 Double ERK1/2–ERK5 targeting in ex vivo patient samples

**FIGURE 6:**
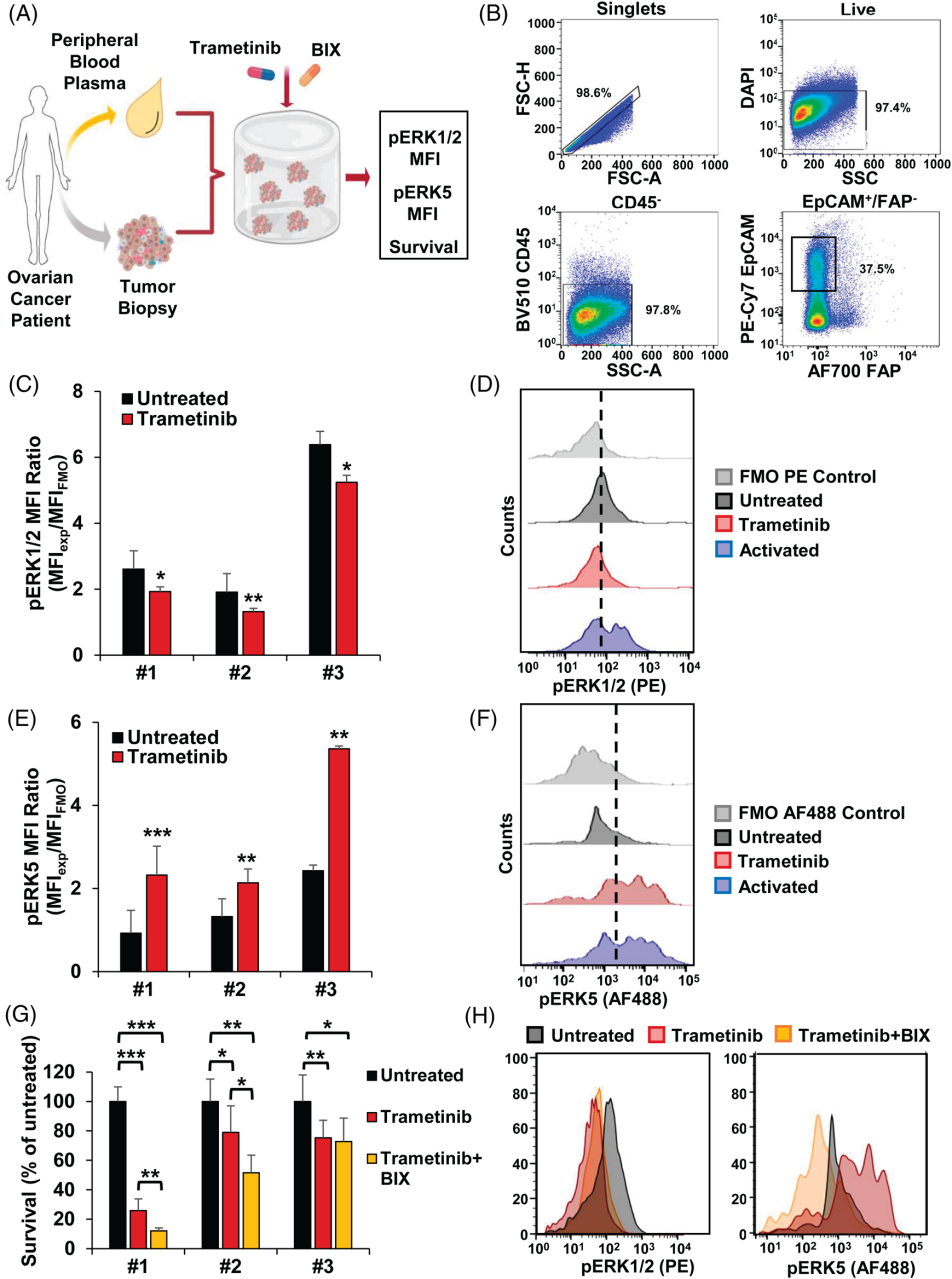
Anti‐tumoural effect of MEK5 and MEK1/2 targeting in a three‐dimensional model utilising patient biopsies. (A) Ovarian cancer patient tumour biopsies and plasma samples were obtained from ovarian cancer patients. Tissue biopsies were enzymatically digested and grown in HuP3D cultures made from the matching patient plasma. HuP3D cultures were treated with trametinib and BIX02189 and further evaluated by flow cytometry in terms of mean fluorescence intensity (MFI) expression of pERK1/2 and pERK5 as well as survival. (B) Gating strategy for analysis of patient biopsy material grown in HuP3D cultures. Data acquisition was completed by collecting information for a specified number of events determined by counting beads. Firstly, cellular populations were isolated from beads and then singlets were gated. After that, the live cell population was identified by live/dead viability marker. Following this, CD45− BV510 cells were identified from the live cell population. Finally, EpCAM+ (CD326+) PE‐Cy7 cells and FAP− AF700 cells were identified from the CD45− population. Fluorescence minus one (FMO) controls were used to set the gating for each population BV510 (CD45), PE‐Cy7 (EpCAM) and AF700 (FAP). (C) pERK1/2 expression by EpCAM+ cells from ovarian cancer patients (#1–3) growing within HuP3D cultures after DMSO control (Untreated) and trametinib treatment, quantified as MFI ratio between PE‐anti‐pERK1/2 and FMO PE control. Results are expressed as mean ± SD of an experiment that was repeated three times. An interquartile range (IQR) was used to remove outliers. *, *p* ≤ .05; **, *p* ≤ .01. (D) Representative flow cytometry histogram of PE pERK1/2 expression of EpCAM+ cells from patient #1 after DMSO control (untreated) and trametinib treatment, with PE FMO and activated controls, as negative and positive controls, respectively. Vertical line is included to indicate the pERK1/2 positive population defined by FMO control. (E) pERK5 expression by EpCAM+ cells from ovarian cancer patients (#1–3) growing within HuP3D cultures after DMSO control (untreated) and trametinib treatment, quantified as MFI ratio between AF488‐anti‐pERK5 and AF488 FMO control. Results are expressed as in (C). (F) Representative flow cytometry histogram of pERK5 expression (AF488) of EpCAM+ cells from patient #1 after DMSO control (untreated) and trametinib treatment, with AF488 FMO and activated controls, as negative and positive controls, respectively. Vertical line is included to indicate the pERK5 positive population defined by FMO control. (G) Effect of DMSO control (untreated), trametinib alone or trametinib in combination with BIX02189 on primary ovarian cancer EpCAM+ cell survival in HuP3D cultures from patients #1 to 3. Results are expressed as in (C). *, *p* ≤ .05; **, *p* ≤ .01. ***, *p* ≤ .001. (H) Representative flow cytometry histogram of pERK1/2 (PE) and pERK5 expressions (AF488) of EpCAM+ cells from patient #2 after DMSO control (untreated), trametinib alone or trametinib in combination with BIX02189.

The potential clinical relevance of these findings led us to explore the hypothesis of targeting ERK5 and ERK1/2 routes in an ex vivo 3D model (HuP3D) that uses ovarian serous cancer cells obtained from patient biopsies, and which are cultured in a 3D matrix made with plasma from the same patient (Figure 6A). To specifically analyse tumour cells, they were isolated and identified by gating cells demonstrating high EPCAM/low FAP signal after dead and immune cell removal as described in the *Methods* section and illustrated in Figure 6B. Thus, patient‐derived HuP3D ovarian cancer cells obtained from three different biopsies (patients #1, #2 and #3) showed that trametinib treatment significantly decreased pERK1/2 expression (Figure 6C). As represented by the flow cytometry histograms, pERK1/2 expression in the DMSO control (untreated) was increased in comparison to the negative control (PE FMO control) and overlaid with the positive control (activated), reflecting the known phosphorylation of ERK1/2 in ovarian cancer.^42^ Treatment with trametinib shifted the EpCAM+ population to the left, indicating a decrease in pERK1/2 expression (Figure 6D). Furthermore, we identified that as pERK1/2 expression was decreased, pERK5 expression was simultaneously and significantly increased for the same three ovarian cancer patients (Figure 6E). Flow cytometry histograms revealed that while pERK5 expression in the DMSO control (untreated) was very minimal as in the negative control (AF488 FMO control), trametinib treatment clearly shifted the EpCAM+ population to the right, indicating an increase in pERK5 expression comparable to the positive control (activated) (Figure 6F). It should be mentioned that the pERK5 antibody could react with pERK1/2 protein in flow cytometry experiments. However, in this case, it should not be a concern since an increase in pERK5 was observed in trametinib‐treated samples, in which pERK1/2 levels were clearly inhibited by the drug.

After confirmation that trametinib treatment activated pERK5 expression as a potential compensatory mechanism, we investigated the effect of BIX02189 in the EpCAM+ population of primary ovarian samples. First, we studied the effect of BIX02189 treatment on pERK1/2 and pERK5 expression in HuP3D cultures from a fourth patient, given the limitation of patient biopsy material from our patients #1–3. While pERK1/2 remained unaltered after BIX02189 treatment, pERK5 expression was clearly reduced when compared to DMSO control (untreated) (Figure S5A). Then, we determined a modest (∼20%) but significant killing effect of BIX02189 treatment when compared with untreated control in the survival of EpCAM+ primary ovarian cancer cells (Figure S5B). Furthermore, we investigated the potential therapeutic benefit of trametinib and BIX02189 combinatory treatment to overcome pERK5 overexpression due to trametinib treatment. Patient‐derived HuP3D cultures identified that combination of trametinib and BIX02189 had a significant enhanced killing effect on EpCAM+ primary ovarian cancer cells in two out of the three patient‐derived HuP3D cultures, as well as confirmed the significant effect on survival of trametinib treatment for the same three ovarian cancer patients (#1–3) compared with untreated control (Figure 6G). Finally, we confirmed the reduced pERK1/2 expression after trametinib compared with untreated control, where BIX02189 combination did not affect pERK1/2 expression, and further validated that the increased pERK5 expression after trametinib treatment was reverted and reduced to lower expression levels than untreated controls (Figure 6H).

## 4 DISCUSSION

In this paper, we have studied the impact of targeting the WNK1–MEK5–ERK5 route in ovarian cancer. Phosphorylation of WNK1 was detected in both patient samples and ovarian cancer cell lines. In the latter, antibody arrays demonstrated that this was the most phosphorylated protein from all the signalling proteins analysed. WNK1 has been involved in certain types of cancer and has been reported to act upstream of the MEK5–ERK5 module,^18^ which has also been implicated in the pathophysiology of various neoplasias. Thus, ERK5 activation through WNK1 and MAP3K2 promoted prostate tumour growth and metastasis^43^ as well as proliferation of human chronic myeloid leukaemia cells.^44^ In the panel of patient samples from our hospital, WNK1 activation had a negative impact on survival. Moreover, in silico analyses in ovarian cancer patients collected from public databases, showed that high expression of WNK1 correlated with poor outcome. Studies by other authors have shown that primary and recurrent post‐chemotherapy ovarian high‐grade serous carcinomas expressed higher levels of WNK1.^45^ WNK1 was found to be hyperphosphorylated in retinoblastoma^46^ and WNK1 phosphosignalling was also identified in breast cancers.^47^

The genetic and pharmacological studies performed pointed to an implication of WNK1 in the control of proliferation of ovarian cancer cells. Moreover, those studies showed a link between WNK1 and the MEK5–ERK5 pathway in ovarian cancer cells. In fact, downregulation of WNK1 or its pharmacological inhibition resulted in a reduction in ERK5 phosphorylation. Furthermore, downregulation of the WNK1 downstream kinases MEKK2, MEK5 and ERK5 caused a reduction in proliferation of ovarian cancer cells. Of note, the down regulation of WNK1 had a more profound effect on cell proliferation than the down regulation of the downstream kinases alone, suggesting that WNK1 may be signalling through pathways other than ERK5. In this respect, WNK1 has been reported to interact with PI3K‐AKT, TGF‐β and NF‐κB signalling in cancer.^48^ The relevance of the MEK5–ERK5 route in the control of ovarian cancer cell proliferation was also supported by the finding that MEK5 KO cells, besides showing inactivation of ERK5, grew much less than wild type parental or Sc OVCAR8 cells, especially in the in vivo setting. Several studies have implicated the MEK5–ERK5 signalling cascade in different cancer types (reviewed in Refs. ^24^ and ^49^). However, to our knowledge, this is the first demonstration of the relevance of the WNK1–MEK5–ERK5 axis in ovarian cancer.

From a translational point of view, our findings may impact in the development of novel strategies to fight ovarian cancer and may also help in refining existing ones. With respect to the development of novel strategies, the potential clinical value of agents that target some of the WNK1–MEK5–ERK5 route components should be explored. Of note, a substantial number of ovarian cancer patients (around 12%) have amplification of *WNK1* and the evaluation of this gene could be used as biomarker for patient selection. Targeting of amplified genes that code for oncogenic kinase proteins has shown successful results in other indications like HER2 in breast cancer.^50^ However, one of the limitations in this field is the lack of effective, specific and potent agents that may act on the components of this route. While compounds that inhibit ERK5 and MEK5 are available for preclinical studies, none of them has entered clinical trials. The MEK5–ERK5 field is anxiously expecting the development of such compounds. On the other hand, our studies may aid in optimising the efficacy of therapeutic regimes that use ERK1/2 inhibitors. In fact, an important finding of the present study was the crosstalk observed between the WNK1–ERK5 and the ERK1/2 routes. The latter were found to be constitutively active in the four ovarian cancer cell lines as well as in samples from ovarian cancer patients. Survival analyses performed in the panel of patients from our hospital indicated that the higher the pERK1/2 expression, the worse the prognosis. These data fall in line with others that reported a negative impact of pERK1/2 on patient outcome in ovarian cancer.^13^, ^51^, ^52^ Moreover, those precedents led to the development of clinical trials to assess the potential benefit of targeting the ERK1/2 route with inhibitors, such as trametinib, that act on such pathway. Importantly, in our hands, that inhibitor abolished pERK1/2 expression but increased the amount of pERK5. Trametinib could activate WNK1–MEK5–ERK5 signalling through membrane receptors in ovarian cancer, without ruling out other described activators (reviewed in Refs. ^53^ and ^54^). Simultaneous inhibition of both signalling pathways caused dephosphorylation of ERK1/2 and ERK5, resulting in a more efficient reduction in cell proliferation than that obtained when individually targeting each route. These results were initially obtained in immortalised cell lines and confirmed using the HuP3D patient‐derived, tumour‐like 3D culture method. It is important to emphasise that primary ovarian cultures in HuP3D models contained ovarian cancer cells as well as accessory tumour microenvironment cellular components from the original tumour sample, recapitulating the in vivo environment of the ovarian cancer cells in a 3D culture made with autologous plasma containing key signalling molecules from the same patient.^35^, ^36^, ^55^ The use of cross‐linked plasma as 3D matrix helps with the development of personalised models, making the HuP3D model highly translationally relevant. HuP3D cultures confirmed previous results obtained using cell lines regarding the increased levels of pERK5 after trametinib treatment and the relevance of combinatory treatment with BIX02189 to compensate this increased expression mechanism. As a note, the required concentrations for trametinib and BIX02189 treatment in HuP3D cultures were higher than the concentrations used in 2D cultures and these requirements have been extensively demonstrated before.^35^, ^56^, ^57^ 3D scaffold models recapitulate in vivo‐like drug resistance and higher doses might be needed in order to see clinical therapeutic efficacies. Some of the reasons leading to the higher drug resistance in 3D scaffolds are the presence of cell–ECM interactions,^58^, ^59^, ^60^ matrix stiffness^61^, ^62^ and concentrations gradients inside 3D scaffolds^63^, ^64^ affecting simultaneously drug resistance.

Some recent reports have shown the existence of a compensatory mechanism between both MAPK pathways in cancer (reviewed in Ref. ^65^). In fact, melanoma cells use the ERK5 pathway to escape ERK1/2 pathway blockade, leading to the reactivation of cell proliferation and acquired resistance.^53^ In colorectal cancer, ERK5 provides a common bypass route in intestinal epithelial cells, which rescues cell proliferation upon abrogation of ERK1/2 signalling.^66^ These reports, together with our findings confirm that the compensatory activation of the ERK5 route upon inhibition of the ERK1/2 pathway may be a general phenomenon. This acquires especial relevance in instances in which inhibitors of the ERK1/2 route are used in the clinic. In this respect, the recent report from the GOG 281/LOGS clinical trial that demonstrated the efficacy of trametinib in ovarian cancer^15^ gives extraordinary value to our findings, since our results raise the possibility of combining trametinib with inhibitors of the ERK5 route to improve the outcome of patients with that disease. While the use of two kinase inhibitors may constitute a limitation due to increased toxicities, examples such as the use of cobimetinib in combination with vemurafenib in BRAF‐V600‐mutated melanoma,^67^, ^68^ sustain the clinical feasibility of such approach.

## 5 CONCLUSIONS

Ovarian cancer is an aggressive disease with poor survival rate. Therefore, identification of molecular entities that may provide new treatment options are needed. The present study uncovers the involvement of WNK1 in ovarian cancer pathophysiology upstream of the MEK5–ERK5 MAPK route and shows the potential therapeutic value of the targeting of this route in that disease. These data may impact in the development of novel strategies to fight ovarian cancer. Another important finding of the present study was the activation of the WNK1–ERK5 axis caused by trametinib, bypassing the anti‐tumoural efficacy of this drug. In the context of a recent clinical trial that showed the benefit of trametinib treatment in ovarian cancer, our studies warn of a potential refinement of trametinib therapy by taking into account the WNK1–ERK5 route in future ovarian cancer precision treatment regimens.

## CONFLICT OF INTEREST STATEMENT

P. P. is the co‐founder of Cellatrix LLC; however, there has been no contribution of the aforementioned entity to the current study. P. P., S. B. and K. C. have a provisional patent application on the HuP3D culture method described in this manuscript. The rest of the authors declare no competing interests.

## FUNDING INFORMATION

AE‐O lab: Instituto de Salud Carlos III (ISCIII) (PI15/01180 co‐founded by ERDF, “A way to make Europe” and PI19/00840 co‐funded by the European Union); AP lab: Ministry of Economy and Competitiveness of Spain (BFU2015‐71371‐R and PID2020‐115605RB‐I00); Instituto de Salud Carlos III through CIBERONC; Junta de Castilla y León (CSI146P20); CRIS Cancer Foundation, ACMUMA, UCCTA and ALMOM. Work carried out in European laboratories receives support from the European Community through the Regional Development Funding Program (ERDF) ‘A way to make Europe’. The work carried out in P. Puente laboratory was supported by an Institutional Development Award (5P20GM103548) from the National Institute of General Medical Sciences and the National Cancer Institute of the National Institutes of Health under Award Number R21CA259158 (USA). Studies performed by P. Puente used Sanford Research Flow Cytometry Core Facilities that are supported in part by a COBRE grant from the National Institutes of Health (5P20GM103548).

## Supporting information

Supporting Information

Supporting Information

Supporting Information

Supporting Information

Supporting Information

Supporting Information

Supporting Information

Supporting Information

## Data Availability

The datasets analysed during the current study are available in the cBioportal (https://www.cbioportal.org/) and Firebrowse (http://firebrowse.org/) repositories and are detailed in the methods section. Any other data can be available upon reasonable request to the corresponding author.

## References

[1] Straubhar A , Chi DS , Long Roche K . Update on the role of surgery in the management of advanced epithelial ovarian cancer. Clin Adv Hematol Oncol. 2020;18(11):723‐731. 33406064

[2] Santaballa A , Barretina P , Casado A , et al. SEOM Clinical Guideline in ovarian cancer (2016). Clin Transl Oncol. 2016;18(12):1206‐1212. 10.1007/s12094-016-1588-8 27905052 PMC5138249

[3] Swisher EM , Lin KK , Oza AM , et al. Rucaparib in relapsed, platinum‐sensitive high‐grade ovarian carcinoma (ARIEL2 Part 1): an international, multicentre, open‐label, phase 2 trial. Lancet Oncol. 2017;18(1):75‐87. 10.1016/s1470-2045(16)30559-9 27908594

[4] Boussios S , Karathanasi A , Cooke D , et al. PARP inhibitors in ovarian cancer: the route to “Ithaca”. Diagnostics (Basel). 2019;9(2):55. 10.3390/diagnostics9020055 31109041 PMC6627688

[5] Burger RA , Brady MF , Bookman MA , et al. Incorporation of bevacizumab in the primary treatment of ovarian cancer. N Engl J Med. 2011;365(26):2473‐2483. 10.1056/NEJMoa1104390 22204724

[6] Coleman RL , Brady MF , Herzog TJ , et al. Bevacizumab and paclitaxel‐carboplatin chemotherapy and secondary cytoreduction in recurrent, platinum‐sensitive ovarian cancer (NRG Oncology/Gynecologic Oncology Group study GOG‐0213): a multicentre, open‐label, randomised, phase 3 trial. Lancet Oncol. 2017;18(6):779‐791. 10.1016/s1470-2045(17)30279-6 28438473 PMC5715461

[7] Hamanishi J , Mandai M , Konishi I . Immune checkpoint inhibition in ovarian cancer. Int Immunol. 2016;28(7):339‐348. 10.1093/intimm/dxw020 27055470

[8] Manzano A , Ocaña A . Antibody‐drug conjugates: a promising novel therapy for the treatment of ovarian cancer. Cancers (Basel). 2020;12(8):2223. 10.3390/cancers12082223 32784819 PMC7464539

[9] Stewart D , Cristea M . Antibody‐drug conjugates for ovarian cancer: current clinical development. Curr Opin Obstet Gynecol. 2019;31(1):18‐23. 10.1097/gco.0000000000000515 30531606

[10] Morrison DK . MAP kinase pathways. Cold Spring Harb Perspect Biol. 2012;4(11):a011254. 10.1101/cshperspect.a011254 23125017 PMC3536342

[11] Savoia P , Fava P , Casoni F , Cremona O . Targeting the ERK signaling pathway in melanoma. Int J Mol Sci. 2019;20(6):1483. 10.3390/ijms20061483 30934534 PMC6472057

[12] Khaddour K , Maahs L , Avila‐Rodriguez AM , Maamar Y , Samaan S , Ansstas G . Melanoma targeted therapies beyond BRAF‐mutant melanoma: potential druggable mutations and novel treatment approaches. Cancers (Basel). 2021;13(22):5847. 10.3390/cancers13225847 34831002 PMC8616477

[13] Burotto M , Chiou VL , Lee JM , Kohn EC . The MAPK pathway across different malignancies: a new perspective. Cancer. 2014;120(22):3446‐3456. 10.1002/cncr.28864 24948110 PMC4221543

[14] Ghanaatgar‐Kasbi S , Khazaei M , Rastgar‐Moghadam A , Ferns GA , Hassanian SM , Avan A . The therapeutic potential of MEK1/2 inhibitors in the treatment of gynecological cancers: rational strategies and recent progress. Curr Cancer Drug Targets. 2020;20(6):417‐428. 10.2174/1568009620666200424144303 32329688

[15] Gershenson DM , Miller A , Brady WE , et al. Trametinib versus standard of care in patients with recurrent low‐grade serous ovarian cancer (GOG 281/LOGS): an international, randomised, open‐label, multicentre, phase 2/3 trial. Lancet. 2022;399(10324):541‐553. 10.1016/s0140-6736(21)02175-9 35123694 PMC8819271

[16] Zhou G , Bao ZQ , Dixon JE . Components of a new human protein kinase signal transduction pathway. J Biol Chem. 1995;270(21):12665‐12669. 10.1074/jbc.270.21.12665 7759517

[17] Chiariello M , Marinissen MJ , Gutkind JS . Multiple mitogen‐activated protein kinase signaling pathways connect the cot oncoprotein to the c‐jun promoter and to cellular transformation. Mol Cell Biol. 2000;20(5):1747‐1758. 10.1128/mcb.20.5.1747-1758.2000 10669751 PMC85357

[18] Xu BE , Stippec S , Lenertz L , et al. WNK1 activates ERK5 by an MEKK2/3‐dependent mechanism. J Biol Chem. 2004;279(9):7826‐7831. 10.1074/jbc.M313465200 14681216

[19] Esparís‐Ogando A , Díaz‐Rodríguez E , Montero JC , Yuste L , Crespo P , Pandiella A . Erk5 participates in neuregulin signal transduction and is constitutively active in breast cancer cells overexpressing ErbB2. Mol Cell Biol. 2002;22(1):270‐275. 10.1128/mcb.22.1.270-285.2002 11739740 PMC134212

[20] Abe J , Kusuhara M , Ulevitch RJ , Berk BC , Lee JD . Big mitogen‐activated protein kinase 1 (BMK1) is a redox‐sensitive kinase. J Biol Chem. 1996;271(28):16586‐16590. 10.1074/jbc.271.28.16586 8663194

[21] Kato Y , Tapping RI , Huang S , Watson MH , Ulevitch RJ , Lee JD . Bmk1/Erk5 is required for cell proliferation induced by epidermal growth factor. Nature. 1998;395(6703):713‐716. 10.1038/27234 9790194

[22] Yan L , Carr J , Ashby PR , Murry‐Tait V , Thompson C , Arthur JS . Knockout of ERK5 causes multiple defects in placental and embryonic development. BMC Dev Biol. 2003;3:11. 10.1186/1471-213x-3-11 14675480 PMC324396

[23] Sánchez‐Fdez A , Ortiz‐Ruiz MJ , Re‐Louhau MF , et al. MEK5 promotes lung adenocarcinoma. Eur Respir J. 2019;53(2):1801327. 10.1183/13993003.01327-2018 30442718 PMC6393765

[24] Stecca B , Rovida E . Impact of ERK5 on the Hallmarks of Cancer. Int J Mol Sci. 2019;20(6):1426. 10.3390/ijms20061426 30901834 PMC6471124

[25] Borges J , Pandiella A , Esparís‐Ogando A . Erk5 nuclear location is independent on dual phosphorylation, and favours resistance to TRAIL‐induced apoptosis. Cell Signal. 2007;19(7):1473‐1487. 10.1016/j.cellsig.2007.01.023 17317102

[26] Sánchez‐Fdez A , Re‐Louhau MF , Rodríguez‐Núñez P , et al. Clinical, genetic and pharmacological data support targeting the MEK5/ERK5 module in lung cancer. NPJ Precis Oncol. 2021;5(1):78. 10.1038/s41698-021-00218-8 34404896 PMC8371118

[27] Ortiz‐Ruiz MJ , Álvarez‐Fernández S , Parrott T , et al. Therapeutic potential of ERK5 targeting in triple negative breast cancer. Oncotarget. 2014;5(22):11308‐11318. 10.18632/oncotarget.2324 25350956 PMC4294347

[28] Esparís‐Ogando A , Díaz‐Rodríguez E , Pandiella A . Signalling‐competent truncated forms of ErbB2 in breast cancer cells: differential regulation by protein kinase C and phosphatidylinositol 3‐kinase. Biochem J. 1999;344:339‐348. Pt 2. 10567214 PMC1220649

[29] Way GP , Rudd J , Wang C , et al. Comprehensive cross‐population analysis of high‐grade serous ovarian cancer supports no more than three subtypes. G3 (Bethesda). 2016;6(12):4097‐4103. 10.1534/g3.116.033514 27729437 PMC5144978

[30] Cerami E , Gao J , Dogrusoz U , et al. The cBio cancer genomics portal: an open platform for exploring multidimensional cancer genomics data. Cancer Discov. 2012;2(5):401‐404. 10.1158/2159-8290.Cd-12-0095 22588877 PMC3956037

[31] Gao J , Aksoy BA , Dogrusoz U , et al. Integrative analysis of complex cancer genomics and clinical profiles using the cBioPortal. Sci Signal. 2013;6(269):pl1. 10.1126/scisignal.2004088 23550210 PMC4160307

[32] Nagy Á , Munkácsy G , Győrffy B . Pancancer survival analysis of cancer hallmark genes. Sci Rep. 2021;11(1):6047. 10.1038/s41598-021-84787-5 33723286 PMC7961001

[33] Gyorffy B , Lánczky A , Szállási Z . Implementing an online tool for genome‐wide validation of survival‐associated biomarkers in ovarian‐cancer using microarray data from 1287 patients. Endocr Relat Cancer. 2012;19(2):197‐208. 10.1530/erc-11-0329 22277193

[34] Esparis‐Ogando A , Ocana A , Rodriguez‐Barrueco R , Ferreira L , Borges J , Pandiella A . Synergic antitumoral effect of an IGF‐IR inhibitor and trastuzumab on HER2‐overexpressing breast cancer cells. Ann Oncol. 2008;19(11):1860‐1869. 10.1093/annonc/mdn406 18641009

[35] Calar K , Plesselova S , Bhattacharya S , Jorgensen M , de la Puente P . Human plasma‐derived 3D cultures model breast cancer treatment responses and predict clinically effective drug treatment concentrations. Cancers (Basel). 2020;12(7):1722. 10.3390/cancers12071722 32610529 PMC7407241

[36] Bhattacharya S , Calar K , Evans C , Petrasko M , de la Puente P . Bioengineering the oxygen‐deprived tumor microenvironment within a three‐dimensional platform for studying tumor‐immune interactions. Front Bioeng Biotechnol. 2020;8:1040. 10.3389/fbioe.2020.01040 33015012 PMC7498579

[37] Domcke S , Sinha R , Levine DA , Sander C , Schultz N . Evaluating cell lines as tumour models by comparison of genomic profiles. Nat Commun. 2013;4:2126. 10.1038/ncomms3126 23839242 PMC3715866

[38] Cook DP , Vanderhyden BC . Ovarian cancer and the evolution of subtype classifications using transcriptional profiling†. Biol Reprod. 2019;101(3):645‐658. 10.1093/biolre/ioz099 31187121

[39] Firebrowse . Accessed http://www.firebrowse.org 2022.

[40] cBioportal for cancer genomics . Accessed http://cbioportal.org 2022.

[41] Kaplan–Meier Plotter . Accessed https://kmplot.com/analysis/ 2022.

[42] Liu S , Zou Q , Chen JP , et al. Targeting enhancer reprogramming to mitigate MEK inhibitor resistance in preclinical models of advanced ovarian cancer. J Clin Invest. 2021;131(20):e145035. 10.1172/jci145035 34464356 PMC8516457

[43] Fulford L , Milewski D , Ustiyan V , et al. The transcription factor FOXF1 promotes prostate cancer by stimulating the mitogen‐activated protein kinase ERK5. Sci Signal. 2016;9(427):ra48. 10.1126/scisignal.aad5582 27165781

[44] Wu K , Nie B , Zhou Q , et al. Effects of WNK1 on human chronic myeloid leukemia K562 cells via MAPK7 phosphorylation and its relative mechanism]. Zhongguo Shi Yan Xue Ye Xue Za Zhi. 2020;28(2):365‐370. 10.19746/j.cnki.issn.1009-2137.2020.02.002 32319364

[45] Jinawath N , Vasoontara C , Jinawath A , et al. Oncoproteomic analysis reveals co‐upregulation of RELA and STAT5 in carboplatin resistant ovarian carcinoma. PLoS One. 2010;5(6):e11198. 10.1371/journal.pone.0011198 20585448 PMC2887843

[46] Selvan LDN , Danda R , Madugundu AK , et al. Phosphoproteomics of retinoblastoma: a pilot study identifies aberrant kinases. Molecules. 2018;23(6):1454. 10.3390/molecules23061454 29914080 PMC6100359

[47] Huang KL , Wu Y , Primeau T , et al. Regulated phosphosignaling associated with breast cancer subtypes and druggability. Mol Cell Proteomics. 2019;18(8):1630‐1650. 10.1074/mcp.RA118.001243 31196969 PMC6682998

[48] Gallolu Kankanamalage S , Karra AS , Cobb MH . WNK pathways in cancer signaling networks. Cell Commun Signal. 2018;16(1):72. 10.1186/s12964-018-0287-1 30390653 PMC6215617

[49] Monti M , Celli J , Missale F , et al. Clinical significance and regulation of ERK5 expression and function in cancer. Cancers (Basel). 2022;14(2):348. 10.3390/cancers14020348 35053510 PMC8773716

[50] Esparis‐Ogando A , Montero JC , Arribas J , Ocana A , Pandiella A . Targeting the EGF/HER ligand‐receptor system in cancer. Curr Pharm Des. 2016;22(39):5887‐5898. 10.2174/1381612822666160715132233 27426127

[51] Wang J , Zhou JY , Wu GS . ERK‐dependent MKP‐1‐mediated cisplatin resistance in human ovarian cancer cells. Cancer Res. 2007;67(24):11933‐11941. 10.1158/0008-5472.Can-07-5185 18089824

[52] Liu S , Zha J , Lei M . Inhibiting ERK/Mnk/eIF4E broadly sensitizes ovarian cancer response to chemotherapy. Clin Transl Oncol. 2018;20(3):374‐381. 10.1007/s12094-017-1724-0 28766096

[53] Benito‐Jardón L , Díaz‐Martínez M , Arellano‐Sánchez N , Vaquero‐Morales P , Esparís‐Ogando A , Teixidó J . Resistance to MAPK inhibitors in melanoma involves activation of the IGF1R‐MEK5‐Erk5 pathway. Cancer Res. 2019;79(9):2244‐2256. 10.1158/0008-5472.Can-18-2762 30833419

[54] Hou CY , Ma CY , Yuh CH . WNK1 kinase signaling in metastasis and angiogenesis. Cell Signal. 2022;96:110371. 10.1016/j.cellsig.2022.110371 35649473

[55] Bhattacharya S , Calar K , de la Puente P . Mimicking tumor hypoxia and tumor‐immune interactions employing three‐dimensional in vitro models. J Exp Clin Cancer Res. 2020;39(1):75. 10.1186/s13046-020-01583-1 32357910 PMC7195738

[56] Hongisto V , Jernström S , Fey V , et al. High‐throughput 3D screening reveals differences in drug sensitivities between culture models of JIMT1 breast cancer cells. PLoS One. 2013;8(10):e77232. 10.1371/journal.pone.0077232 24194875 PMC3806867

[57] Choudhury P , Gupta M . Personalized & precision medicine in cancer: a theranostic approach. Curr Radiopharm. 2017;10(3):166‐170. 10.2174/1874471010666170728094008 28758574

[58] Holle AW , Young JL , Spatz JP . In vitro cancer cell‐ECM interactions inform in vivo cancer treatment. Adv Drug Deliv Rev. 2016;97:270‐279. 10.1016/j.addr.2015.10.007 26485156

[59] Holohan C , Van Schaeybroeck S , Longley DB , Johnston PG . Cancer drug resistance: an evolving paradigm. Nat Rev Cancer. 2013;13(10):714‐726. 10.1038/nrc3599 24060863

[60] Muranen T , Selfors LM , Worster DT , et al. Inhibition of PI3K/mTOR leads to adaptive resistance in matrix‐attached cancer cells. Cancer Cell. 2012;21(2):227‐239. 10.1016/j.ccr.2011.12.024 22340595 PMC3297962

[61] Hynes RO . Stretching the boundaries of extracellular matrix research. Nat Rev Mol Cell Biol. 2014;15(12):761‐763. 10.1038/nrm3908 25574535

[62] Rice AJ , Cortes E , Lachowski D , et al. Matrix stiffness induces epithelial‐mesenchymal transition and promotes chemoresistance in pancreatic cancer cells. Oncogenesis. 2017;6(7):e352. 10.1038/oncsis.2017.54 28671675 PMC5541706

[63] de la Puente P , Muz B , Gilson RC , et al. 3D tissue‐engineered bone marrow as a novel model to study pathophysiology and drug resistance in multiple myeloma. Biomaterials. 2015;73:70‐84. 10.1016/j.biomaterials.2015.09.017 26402156 PMC4917006

[64] Tannock IF , Lee CM , Tunggal JK , Cowan DS , Egorin MJ . Limited penetration of anticancer drugs through tumor tissue: a potential cause of resistance of solid tumors to chemotherapy. Clin Cancer Res. 2002;8(3):878‐884. 11895922

[65] Tubita A , Tusa I , Rovida E . Playing the whack‐a‐mole game: eRK5 activation emerges among the resistance mechanisms to RAF‐MEK1/2‐ERK1/2‐targeted therapy. Front Cell Dev Biol. 2021;9:647311. 10.3389/fcell.2021.647311 33777953 PMC7991100

[66] de Jong PR , Taniguchi K , Harris AR , et al. ERK5 signalling rescues intestinal epithelial turnover and tumour cell proliferation upon ERK1/2 abrogation. Nat Commun. 2016;7:11551. 10.1038/ncomms11551 27187615 PMC4873670

[67] Ascierto PA , Dreno B , Larkin J , et al. 5‐Year outcomes with cobimetinib plus vemurafenib in BRAFV600 mutation‐positive advanced melanoma: extended follow‐up of the coBRIM study. Clin Cancer Res. 2021;27(19):5225‐5235. 10.1158/1078-0432.CCR-21-0809 34158360 PMC9401485

[68] Ascierto PA , McArthur GA , Dreno B , et al. Cobimetinib combined with vemurafenib in advanced BRAF(V600)‐mutant melanoma (coBRIM): updated efficacy results from a randomised, double‐blind, phase 3 trial. Lancet Oncol. 2016;17(9):1248‐1260. 10.1016/S1470-2045(16)30122-X 27480103

