# Supplementary material for: The WNK1–ERK5 route plays a pathophysiological role in ovarian cancer and limits therapeutic efficacy of trametinib
Source: Clin Transl Med. 2023 Apr 8;13(4):e1217. doi: 10.1002/ctm2.1217 (PMC10082568; doi:10.1002/ctm2.1217)
Supplement: Supplementary file 6 — Supporting Information [file CTM2-13-e1217-s003.docx]

**Supplementary Figure 1. pWNK1, WNK1, pERK1/2 and ERK1/2 expression in a cohort of ovarian cancer patients from our hospital.** Western blot analyses of pWNK1, WNK1, pERK1/2 and ERK1/2 expression in 63 epithelial ovarian cancer samples, including a nontumoral sample (N), obtained from 57 patients from the University Hospital of Salamanca. Numbers correspond to the tissue bank classification of each patient. 70 µg (pWNK1 and WNK1), 80 µg (pERK1/2) or 40 µg (ERK1/2) of protein extract were analyzed by Western blotting with the corresponding antibodies. Calnexin was used as loading control. Several bands, which may be due to the existence of WNK1 isoforms, are observed in the WNK1 and pWNK1 blots..

**Supplementary Figure 2. *WNK1* and patient outcome.** A) Overall survival curves of those ovarian serous carcinoma patients from the TCGA Firehose Legacy dataset with available Agilent microarray mRNA expression and clinical data (n=533). Patients harboring WNK1 mRNA upregulated levels (red line) were compared to those without such alteration (blue line). The *p-*value of the study, follow-up (months) and median overall survival of each cohort are indicated. B) 48 months follow-up Kaplan-Meier analysis of the relationship between WNK1 expression levels and Post-Progression Survival (n=735) or C) Progression-Free Survival (n=1104) in ovarian cancer patients collected in the mRNA gene chip section of the Kaplan-Meier plotter database. Patients were stratified according to low or high WNK1 expression by selecting the best cutoff automatic tool as indicated in the Materials and Methods section. The *p-*value, hazard ratio, False Discovery Rate (FDR), median overall survival, and number of patients at risk in the low and high expression groups are indicated.

**Supplementary Figure 3. Effect of trametinib on the activation of WNK1-MEK5- ERK5 axis.** A) OVCAR8 cells were treated with 50 nM trametinib for the indicated times and immediately lysed. Western blotting of 60 µg of whole-cell lysates were used to detect pERK1/2 along time. B) OVCAR8 cells were treated with 5µM BIX02189, 50 nM trametinib or both for 4 hours and immediately lysed. 80 µg (ERK5) or 40 µg (pERK1/2) of whole-cell lysates were used to detect these proteins by Western blotting with their corresponding antibodies. Calnexin was used as loading control. C) OVCAR8 cells were either untreated or treated with 50 nM trametinib for 4 hours and immediately lysed. While 70 µg of whole-cell lysates were used to detect pWNK1 and MEK5 by Western blotting, 1mg of protein extracts were immunoprecipitated with the ERK5 or MEK5 antibodies and detected by Western blotting with the pMEK5 or pERK5 antibodies, respectively. Calnexin was used as loading control. D) A2780 and SKOV3 cells were treated as in (C) and pERK1/2 and ERK5 expression were evaluated by Western blotting with the pERK1/2 or ERK5 antibodies, respectively. E) OVCAR8 cells were treated with increasing doses of trametinib for 4 hours and lysed. Then ERK5 was immunoprecipitated and subjected to the in vitro kinase assay as described in the Materials and Methods section. The reaction was started by ATP addition. pERK5 forms were detected by Western Blotting. F) OVCAR8 cells were treated with the indicated doses of WNK463 or BIX02189 for 4 hours and immediately lysed. 80 to 40 µg of whole-cell lysates were used to detect ERK1/2, pERK1/2 and ERK5 expression by Western blotting with their corresponding antibodies. GAPDH was used as loading control. G) OVCAR8, SKOV3 and A2780 cells were plated in p6 wells and treated with the indicated doses of WNK463 and trametinib (individually and combined), for 3 days. Cell proliferation was measured by cell counting and represented as percentage from control untreated cells. Data is presented as the mean ± SD of an experiment that was repeated three times. **, p≤0.01; ***, p≤0.001. H) OVCAR8 pLKO shControl cells and OVCAR8 MEK5 knockdown cells (sh66 and sh70) were plated in 24-well dishes, and

24 hours later treated with the indicated doses of trametinib for 2 days. Cell proliferation was measured by an MTT assay, and each condition represented as percentage from its respective untreated cells. Results are expressed as mean ± SD of an experiment that was repeated twice. *, p≤0.05; **, p≤0.01. I) The MEK5 CRISPR tumors from the vehicle and trametinib treated mice were resected and processed as described in the material and methods section. 100 µg of protein extract were used to detect pERK1/2 and ERK5 by Western blotting. Calnexin was used as loading control.

**Supplementary Figure 4. Response to trametinib treatment in mice xenografted with Sc and MEK5 CRISPR cells.** Mice were xenografted with OVCAR8 Sc or MEK5 CRISPR cells at day 0 in the graph. Once tumors reached a mean volume of approximately 500mm3, mice were treated with vehicle or trametinib (100 µL of trametinib 0.5 mg/Kg,

i.p. daily for five weeks). Tumor volumes of Sc and MEK5 CRISPR groups were measured weekly. Note the treatment in the mice injected with MEK5 CRISPR cells started almost one month later than in the case of the mice injected with OVCAR8 Sc cells. p-values are indicated.

**Supplementary Figure 5. Effect of MEK5 inhibition on the survival of primary ovarian cancer cells in a 3D ex vivo human model**. A) Representative flow cytometry histogram of pERK1/2 (PE) and pERK5 (AF488) expressions of EpCAM+ cells from patient #4 after DMSO control (Untreated) and BIX02189 treatment. B) Effect of BIX02189 treatment on primary ovarian cancer EpCAM+ cell survival in HuP3D cultures from patient #4, compared to DMSO control (Untreated). **, p≤0.01.
