# Supplementary material for: The WNK1–ERK5 route plays a pathophysiological role in ovarian cancer and limits therapeutic efficacy of trametinib
Source: Clin Transl Med. 2023 Apr 8;13(4):e1217. doi: 10.1002/ctm2.1217 (PMC10082568; doi:10.1002/ctm2.1217)
Supplement: Supplementary file 7 — Supporting Information [file CTM2-13-e1217-s004.docx]

**Supplementary Table 1. Association of pWNK1 with patient survival using a multivariate cox-regression analysis including clinicopathological characteristics.**

| **Variable** | **HR (95% CI)** | **p-value** |
| --- | --- | --- |
| pWNK1 | 1.3 (1.1-1.6) | .0005 |
| Age | 1.1 (1-1.1) | .013 |
| Histology | 0.39 (0.14-1.1) | .072 |
| Grade | 1 (0.31-3.3) | .99 |
| Size | 0.99 (0.89-1.1) | .9 |
| Stage | 1.4 (0.66-2.9) | .4 |
