# Supplementary material for: The WNK1–ERK5 route plays a pathophysiological role in ovarian cancer and limits therapeutic efficacy of trametinib
Source: Clin Transl Med. 2023 Apr 8;13(4):e1217. doi: 10.1002/ctm2.1217 (PMC10082568; doi:10.1002/ctm2.1217)
Supplement: Supplementary file 8 — Supporting Information [file CTM2-13-e1217-s006.docx]

**Supplementary Table 2. Association of pERK1/2 with patient survival using a multivariate cox-regression analysis including clinicopathological characteristics.**

| **Variable** | **HR (95% CI)** | **p-value** |
| --- | --- | --- |
| pErk1/2 | 1.2 (1.1-1.3) | 5.6e-06 |
| Age | 1 (1-1.1) | .017 |
| Histology | 0.64 (0.3-1.4) | .24 |
| Grade | 0.45 (0.097-2.1) | .31 |
| Size | 0.95 (0.85-1.1) | .40 |
| Stage | 0.85 (0.44-1.7) | .63 |
